# Pregestational Cardiometabolic Biomarkers and Future Hypertensive Disorders of Pregnancy

**DOI:** 10.1001/jamanetworkopen.2026.10037

**Published:** 2026-04-30

**Authors:** Angelika Qvick, Anna Sandström, Anna Norhammar, Max Vikström, Anna-Clara Spetz Holm, Rebecka Hultgren, Niklas Hammar, Karin Leander

**Affiliations:** 1 Unit of Cardiovascular and Nutritional Epidemiology, Institute of Environmental Medicine, Karolinska Institutet, Stockholm, Sweden; 2 Department of Medicine, Solna, Clinical Epidemiology Division, Karolinska Institutet, Stockholm, Sweden; 3 Department of Obstetrics and Gynecology, Karolinska University Hospital, Stockholm, Sweden; 4 Department of Medicine, Karolinska Institutet, Karolinska University Hospital, Stockholm, Sweden; 5 Capio St Görans Hospital, Stockholm, Sweden; 6 Department of Biomedical and Clinical Sciences, Linköping University, Linköping, Sweden; 7 Clinical Department of Obstetrics and Gynaecology in Linköping, Region Östergötland, Linköping, Sweden; 8 Department of Molecular Medicine and Surgery, Karolinska Institutet, Stockholm, Sweden; 9 Department of Vascular Surgery, Karolinska University Hospital, Stockholm, Sweden; 10 Unit of Epidemiology, Institute of Environmental Medicine, Karolinska Institutet, Stockholm, Sweden

## Abstract

**Importance:**

Hypertensive disorders of pregnancy (HDP) are one of the leading causes of maternal morbidity and death. There is an urgent need to improve early identification of women at risk of HDP, and assessment of pregestational cardiometabolic biomarkers is a potential way forward.

**Objective:**

To investigate pregestational cardiometabolic disturbances with regard to risk of HDP.

**Design, Setting, and Participants:**

This prospective cohort study used data from the Apolipoprotein-Related Mortality Risk (AMORIS) cohort, a population-based study set in the greater Stockholm area of Sweden, linked to the Swedish Medical Birth Register from January 1, 1985, to December 31, 2020. Participants were nulliparous women 18 years of age or older with blood biomarker data obtained before their first completed pregnancy. Data analyses were conducted from January 1 to October 31, 2025.

**Exposures:**

Pregestational cardiometabolic disturbances, identified through biomarkers of lipid and glucose metabolism and low-grade inflammation. Median time between blood sampling and the start of pregnancy was 4 to 6 years (range, 0-31 years).

**Main Outcomes and Measures:**

HDP, defined as gestational hypertension, preeclampsia, or superimposed preeclampsia.

**Results:**

Of 35 189 women included (mean [SD] age at delivery, 30.9 [4.8] years), 1938 (5.5%) had HDP. In groups identified with pregestational cardiometabolic disturbances, the percentages with HDP were between 5.5% and 12.8%, whereas the percentages in the comparison categories were between 4.1% and 5.3%. In multivariable logistic regression models, associations with increased risk of HDP were observed for quartile (Q) 4 (vs Q1) for total cholesterol (adjusted odds ratio [AOR], 1.23 [95% CI, 1.06-1.41]), low-density lipoprotein cholesterol (AOR, 1.41 [95% CI, 1.05-1.89]), triglycerides (AOR, 1.19 [95% CI, 1.03-1.37]), haptoglobin (AOR, 1.20 [95% CI, 1.02-1.42]), apolipoprotein (Apo) B (AOR, 1.90 [95% CI, 1.36-2.65]), ApoB/ApoA1 ratio (AOR, 1.59 [95% CI, 1.10-2.30]), and the triglyceride-glucose index (AOR, 1.21 [95% CI, 1.04-1.40]). For C-reactive protein (AOR, 0.97 [95% CI, 0.80-1.17]) and leukocyte counts (AOR, 0.98 [95% CI, 0.80-1.20]), there was no association with HDP risk.

**Conclusions and Relevance:**

In this cohort study, pregestationally assessed cardiometabolic biomarkers were associated with increased risk of HDP in nulliparous women. For some biomarkers, the increased risk was observed below standard cutoff levels for a clinical diagnosis, that is, at subclinical levels. These results suggest that assessment of cardiometabolic biomarkers may improve identification of women at risk of HDP, both in preconceptional counseling settings and at enrollment in antenatal health care.

## Key Points

**Question:**

Are pregestational cardiometabolic disturbances associated with risk of hypertensive disorders of pregnancy (HDP)?

**Findings:**

In this cohort study including 35 189 nulliparous women, pregestationally assessed cardiometabolic biomarkers were associated with an increased risk of HDP. For some lipid and glucose biomarkers, subclinical levels (below established clinical thresholds) were associated with an increased HDP risk for a future pregnancy.

**Meaning:**

These results may contribute to early identification of women at risk of HDP and suggest a need for the use of cardiometabolic biomarkers in preconceptional counseling and at enrollment in antenatal care.

## Teaser

This cohort study investigates whether pregestational cardiometabolic disturbances identified through biomarkers of lipid and glucose metabolism and low-grade inflammation are associated with the risk of future hypertensive disorders of pregnancy.

## Introduction

Hypertensive disorders of pregnancy (HDP), including chronic hypertension, gestational hypertension, preeclampsia, and superimposed preeclampsia, affect 5% to 15% of pregnancies^1,2,3,4,5^ and are major causes of maternal and neonatal morbidity and mortality.^6,7,8^ HDP also pose a long-term, sex-specific risk for future cardiovascular disease (CVD).^9^ In recent years, increasing incidence rates of HDP have been observed,^5,10^ most likely attributable to an increasing mean maternal age at first delivery and an increasing prevalence of obesity.^11,12^

The pathophysiology of HDP remains incompletely understood.^4^ Current evidence suggests that HDP arise from maladaptive cardiovascular responses to the physiological demands of pregnancy.^13^ Such responses may include impaired vascular adaptation, which is implicated in the development of preeclampsia.^4,14^ Established early pregnancy risk factors include advanced maternal age, obesity, and chronic conditions such as diabetes, and hypertension.^15,16^ Most clinical guidelines rely on data collected during or after pregnancy, limiting opportunities for early intervention. To enhance prevention strategies, there is a critical need to identify risk factors detectable before conception or in early pregnancy, when preventive measures, such as lifestyle changes, may be more effective.

Women with HDP often exhibit greater dyslipidemia,^17,18^ impaired fasting glucose,^19^ and elevated inflammatory biomarkers^20^ during or after pregnancy compared with normotensive counterparts. Whether these cardiometabolic disturbances are present before conception remains unclear. A limited number of studies suggest that elevated fasting glucose,^21,22,23^ and triglyceride^22,23,24^ levels and reduced high density lipoprotein cholesterol (HDL-C)^22,23^ before pregnancy may be associated with increased HDP risk, but opposite findings have also been reported.^25,26^ Variability in study design, such as inclusion of multiparous women or differing approaches to clinical biomarker thresholds, may partly explain these discrepancies. Given the prevalence and adverse outcomes of HDP, a stronger evidence base for early prevention is needed. The aim of this study was to investigate pregestational cardiometabolic disturbances, assessed through biomarkers of lipid and glucose metabolism and low-grade inflammation, and risk of HDP in nulliparous women. Both standard clinically used and subclinical cutoffs were considered.

## Methods

This prospective cohort study was approved by the Regional Ethical Review Board in Stockholm, Sweden, which waived the requirement of informed consent mainly due to the long interval since the baseline examination. It was conducted in coherence with Strengthening the Reporting of Observational Studies in Epidemiology (STROBE) reporting guideline.^27^ The study used data from the Swedish Apolipoprotein-Related Mortality Risk (AMORIS) cohort, which comprised 812 073 residents of Sweden, primarily from the greater Stockholm area, referred to laboratory testing as a part of routine health examinations in occupational or primary care settings from 1985 through 1996.^28^

This study included nulliparous women aged at least18 years at the start of their first completed pregnancy (index pregnancy) between January 1, 1985, and December 31, 2020. Eligible participants had at least 1 pregestational biomarker measurement reflecting cardiometabolic status and complete data on selected covariates. Pregnancy data were obtained by linking AMORIS to the Swedish Medical Birth Register (MBR)^29^ using unique personal identification numbers. Completed pregnancies were defined as those ending after 28 or more weeks of gestation (before July 1, 2008) or 22 or more weeks of gestation (as of July 1, 2008). Estimated start of the index pregnancy was computed based on gestational length at birth and date of birth. Gestational length was defined hierarchically by ultrasound in first or second trimester, date of last menstrual period, or estimated gestational length in the obstetric clinical record.

A total of 35 189 individuals met the inclusion criteria (eFigure 1 in Supplement 1). The mean age at inclusion in AMORIS (42.6 years) meant that a large proportion of women had already given birth to their first child at the time of sampling and thus did not meet eligibility criteria. The median time between biomarker sampling and the estimated start of the index pregnancy varied from 4 to 6 years; the range varied from 0 to 31 years (eTable 1 in Supplement 1). Participants were followed up from antenatal care registration until HDP diagnosis or end of follow-up (1 week postpartum).

Participant characteristics were obtained through linkage with the MBR, the National Patient Register (NPR),^30^ the National Prescribed Drug Register,^31^ the Longitudinal Integrated Database for Health Insurance and Labor Market Studies,^32^ and the National Diabetes Register^33^ (eTable 2 in Supplement 1). Early pregnancy body mass index (BMI) was categorized per World Health Organization (WHO) definitions^34^ and calculated as weight in kilograms divided by height in meters squared from weight and height recorded before 13 weeks’ gestation in the MBR (available for >90% of pregnancies). Maternal weight data were unavailable in the 1990-1991 period. For this period, BMI data from a subsequent pregnancy were used; if unavailable, an obesity diagnosis recorded in the MBR or NPR before delivery was used. Use of antihypertensive drugs before the index pregnancy was considered. Maternal chronic conditions before 20 weeks’ gestation were considered. Chronic hypertension included diagnoses of chronic hypertension or gestational hypertension (GH) in the MBR or NPR. Diabetes and its subtypes were identified hierarchically via the NDR, the MBR or NPR, or dispensed antidiabetic drugs. Known dyslipidemia was defined by diagnosis in the MBR or NPR or lipid-lowering medication use. History of diagnosed polycystic ovarian syndrome, systemic lupus erythematosus, chronic kidney disease, antiphospholipid syndrome, and CVD was obtained from the MBR or NPR. Educational level before pregnancy was categorized into 4 groups: no mandatory education, primary, secondary, or university. Use of cigarettes and snuff was assessed via antenatal interviews at 3 time points and categorized as ever or never use. Maternal and infant antenatal and perinatal characteristics were obtained from the MBR or NPR. Further details on the assessment of characteristics are provided in eTable 3 in Supplement 1.

### Assessment of Pregestational Cardiometabolic Disturbances

Pregestational cardiometabolic disturbances were assessed using biomarkers from AMORIS health examinations. Lipid metabolism biomarkers included apolipoprotein A1 (ApoA1), apolipoprotein B (ApoB), the ApoB/ApoA1 ratio, low-density lipoprotein cholesterol (LDL-C), HDL-C, non–HDL-C, fasting triglycerides, and total cholesterol (TC). Glucose metabolism biomarkers were fasting glucose and the triglyceride-glucose (TyG) index. Biomarkers of inflammation included nonsensitive C-reactive protein (CRP), haptoglobin, and the leukocyte count. Biomarkers were selected based on clinical relevance and data availability (eTable 4 in Supplement 1). The samples obtained closest to the index pregnancy were used when multiple measurements existed. Analyses were performed consistently using automated methods described elsewhere^35,36,37^ and in the eMethods in Supplement 1.

In this study, the term subclinical cutoff is used for levels that did not reach the threshold for a clinical diagnosis according to current guidelines. The biomarkers were categorized based on subclinical and, when relevant, clinical cutoffs. Such relevance was assessed based on whether the biomarker has widespread use for diagnosing dyslipidemia or diabetes. For biomarkers with clinical cutoffs (fasting glucose, fasting triglycerides, LDL-C, HDL-C, non–HDL-C, and TC), 4 separate categories were created: dyslipidemia; prediabetes according to the American Diabetes Association (ADA); prediabetes according to WHO; and diabetes. Individuals with values above the cutoff for a clinical diagnosis or with a known diagnosis of diabetes or dyslipidemia were placed in the respective category. Age-specific clinical cutoffs were used for fasting triglycerides (>248 mg/dL [1-17 years] and >230 mg/dL [≥18 years]; to convert to mmol/L, multiply by 0.0113), TC (>228 mg/dL [1-14 years], >232 mg/dL [15-17 years], >236 mg/dL [18-30 years], and >267 mg/dL [31-50 years]), LDL-C (>155 mg/dL [1-17 years], >166 mg/dL [18-30 years], >182 mg/dL [31-50 years]), and non–HDL-C (>201 mg/dL [<1 year], >166 mg/dL [1-9 years], >155 mg/dL [10-17 years], >182 mg/dL [18-29 years], >197 mg/dL [30-49 years], >240 mg/dL [>50 years]) (to convert LDL-C, non–HDL-C, and TC to mmol/L, multiply by 0.0259) (eTable 6 in Supplement 1).For the analyses of subclinical levels, quartiles (Q) were used, with Q1 serving as the reference, except for the analyses of HDL-C and ApoA1, for which Q4 served as the reference. The separate clinical categories were mutually exclusive from quartile categories. Inclusion in a clinical category meant exclusion from the quartile classification.

### Outcome

The outcome was a diagnosis of any HDP, including GH, preeclampsia, or superimposed preeclampsia, occurring from 20 weeks’ gestation until 1 week postpartum, excluding isolated chronic hypertension. HDP were identified through primary or secondary *International Classification of Diseases (ICD)*-coded diagnoses in the MBR or NPR for inpatient care, at least 2 primary or secondary *ICD*-coded diagnoses in the NPR for outpatient visits, or dispensed antihypertensive drugs for pregnancy-induced hypertension from 20 weeks’ gestation onward (eTable 3 in Supplement 1). Diagnostic criteria for HDP in Sweden follow international classifications.^38^ Since 2019, preeclampsia requires new-onset hypertension (systolic/diastolic blood pressure ≥140/90 mm Hg) after 20 weeks’ gestation plus proteinuria or organ involvement (eg, fetal growth restriction); prior to 2019, proteinuria was mandatory. GH was defined as new-onset hypertension after 20 weeks’ gestation.

### Statistical Analysis

Statistical analyses were conducted from January 1 to October 31, 2025, and used Stata IC, version 16.0, Stata SE (both from StataCorp LLC), and SAS, version 9.4 (SAS Institute Inc). Descriptive statistics included means and SDs for normally distributed variables, medians and IQRs for skewed variables, and frequencies for categorical data. Standardized mean differences (SMDs) were calculated to assess differences.^39^ Associations between biomarkers and HDP risk were estimated using logistic regression, with odds ratios (ORs) and 95% CIs reported from crude and multivariable models. A complete-case approach was applied. A 95% CI excluding 1.00 was considered statistically significant.

Potential confounders included early pregnancy BMI (categorical, with normal weight as reference), maternal age at delivery, calendar year of index pregnancy, and chronic hypertension. Analyses of inflammatory and lipid biomarkers were additionally adjusted for pregestational diabetes, whereas analyses involving inflammatory and glucose biomarkers were adjusted for dyslipidemia.

Predefined sensitivity analyses included stratification by time between blood sampling and the index pregnancy using 3 categories based on biomarker-specific time tertiles; for TyG index and non–HDL-C levels that were calculated based on measurements of 2 other biomarkers, the time was calculated from the first measured biomarker. A second sensitivity analysis included additional adjustments for polycystic ovarian syndrome, smoking, and BMI as a continuous variable. The third sensitivity analysis was restriction to the following subgroups: occupational health care referrals, singleton pregnancies, and no prophylactic prenatal acetylsalicylic acid use.

To characterize the shape of the associations between continuous biomarker variables and HDP, we used multivariable logistic regression models incorporating restricted cubic splines (knots at the 5th, 27.5th, 50th, 72.5th, and 95th percentiles), allowing for flexible modeling of potential nonlinear relationships. The choice of number of knots and their placement was based on size of study population and number of cases, as recommended.^40^ The models estimated the probability of HDP across the range of biomarker values. For participants with a diagnosis of dyslipidemia or diabetes but missing a registered value for the relevant biomarker, we imputed the median value among participants with biomarker levels above the clinical cutoff. For biomarkers showing linearity in the restricted cubic spline models, we performed additional linear regressions.

## Results

The number of women eligible for inclusion was 35 189 (mean [SD] age at delivery, 30.9 [4.8] years). Of them, 1938 (5.5%) were diagnosed with HDP during their index pregnancy. In groups identified with cardiometabolic disturbances, the percentages with HDP were between 5.5% and 12.8%, whereas the percentages in the comparison categories were between 4.1% and 5.3%. Compared with normotensive women, women with HDP were slightly older, more frequently overweight or obese, and had higher prevalence of chronic conditions, including diabetes, chronic hypertension, kidney disease, dyslipidemia, polycystic ovarian syndrome, and CVD. Smoking was less common among women with HDP, whereas educational level and snuff use showed no clear differences. HDP pregnancies were more often conceived through assisted reproduction and had multifetal gestation, gestational diabetes, and shorter gestational length. Vaginal delivery was less frequent in the HDP group. Of the HDP cases, 253 (13.1%) were diagnosed with GH and the remainder with preeclampsia. In 22.0% of those with preeclampsia, the condition occurred before full-term pregnancy (37 weeks’ gestation) (Table 1).

**Table 1.** Participant Characteristics

Characteristic	Participants, No. (%)			SMD (normotensive vs HDP pregnancies)
	Complete cohort (N = 35 189)	Normotensive pregnancies (n = 33 251)	HDP pregnancies (n = 1938)	
Age at delivery, y				
<30	14 076 (40.0)	13 376 (40.2)	700 (36.1)	−0.09
30-34	13 179 (37.4)	12 530 (37.6)	649 (33.5)	−0.09
35-39	6310 (17.9)	5879 (17.7)	431 (22.2)	0.11
≥40	1624 (4.6)	1466 (4.4)	158 (8.2)	0.16
BMI in early pregnancy^a^				
Underweight (<18.5)	1159 (3.3)	1114 (3.4)	45 (2.3)	−0.05
Normal weight (18.5-24.9)	25 594 (72.7)	24 476 (73.6)	1118 (57.7)	−0.35
Overweight (25-29.9)	6505 (18.5)	5992 (18.0)	513 (26.5)	0.21
Obesity (≥30)	1931 (5.5)	1669 (5.0)	262 (13.5)	0.29
Educational level,				
No completed mandatory education	77 (0.2)	70 (0.2)	7 (0.4)	0.03
Elementary school	7800 (22.2)	7330 (22.0)	470 (24.3)	0.05
High school	16 253 (46.2)	15 333 (46.1)	920 (47.5)	0.03
University	6879 (19.5)	6496 (19.5)	383 (19.8)	0.001
Missing	4180 (11.9)	4022 (12.0)	158 (8.2)	−0.13
Smoking	5175 (14.7)	4947 (14.9)	228 (11.8)	−0.09
Missing	1967 (5.6)	1847 (5.6)	120 (6.2)	0.03
Snuff use	501 (1.4)	469 (1.4)	32 (1.7)	0.02
Missing	20 780 (59.1)	19 782 (59.5)	998 (51.5)	−0.05
Maternal diagnosis				
Diabetes	865 (2.5)	739 (2.2)	126 (6.5)	0.21
Type 1	94 (10.9)^b^	71 (9.6)^b^	23 (18.3)^b^	
Type 2	242 (28.0)^b^	121 (16.4)^b^	21 (16.7)^b^	
Unspecified	529 (61.2)^b^	547 (74.0)^b^	82 (65.1)^b^	
Chronic hypertension	143 (0.4)	114 (0.3)	29 (1.5)	0.12
Chronic kidney disease	372 (1.1)	318 (1.0)	54 (2.8)	0.14
Dyslipidemia	677 (1.9)	575 (1.7)	1.02 (5.3)	0.15
Systemic lupus erythematosus	49 (0.1)	44 (0.1)	5 (0.3)	0.02
Antiphospholipid syndrome	0 (0)	0 (0)	0 (0)	N/A
Polycystic ovarian syndrome	553 (1.6)	497 (1.5)	56 (2.9)	0.06
Cardiovascular disease	2494 (7.1)	2329 (7.0)	165 (8.5)	0.06
Rheumatic heart disease	23 (0.1)	21 (0.1)	2 (0.1)	0.01
Ischemic heart disease	25 (0.1)	23 (0.1)	2 (0.1)	0.01
Pulmonary heart disease	298 (0.8)	274 (0.8)	24 (1.2)	0.02
Other heart disease	1758 (5.0)	1653 (5.0)	105 (5.4)	0.06
Cerebrovascular disease	454 (1.3)	412 (1.2)	42 (2.2)	0.03
Artery disease	127 (0.4)	117 (0.4)	10 (0.5)	0.01
Pregestational maternal antihypertensive treatment	235 (0.7)	192 (0.6)	43 (2.2)	0.14
Assisted reproduction				
Ovary stimulation	561 (1.6)	524 (1.6)	37 (1.9)	0.03
In vitro fertilization	1493 (4.2)	1358 (4.1)	135 (7.0)	0.13
Singleton or multifetal pregnancy				
Singleton	34 540 (98.2)	32 701 (98.3)	1839 (94.9)	−0.19
Multifetal	649 (1.8)	550 (1.7)	99 (5.1)	0.19
Gestational diabetes	174 (0.5)	151 (0.5)	23 (1.2)	0.08
Gestational length at delivery, median (IQR), d	281 (273-288)	281 (273-288)	275 (263-284)	−0.47
Preterm birth				
Extremely preterm (<28 weeks’ gestation)	94 (0.3)	77 (0.2)	17 (0.9)	0.08
Very preterm (28-32 weeks’ gestation)	245 (0.7)	181 (0.5)	64 (3.3)	0.20
Preterm (>32-37 weeks’ gestation)	1875 (5.3)	1573 (4.7)	302 (15.6)	0.37
Vaginal delivery	21 510 (61.1)	20 721 (62.3)	789 (40.7)	−0.44
Stillbirth	138 (0.4)	132 (0.4)	6 (0.3)	−0.01
Infant birthweight, mean (SD), g	3453.0 ± 573.4	3471.5 ± 552.3	3135.8 ± 795.0	−0.42

Abbreviations: BMI, body mass index; HDP, hypertensive disorders of pregnancy; SMD, standardized mean difference.

^a^ BMI was calculated as weight in kilograms divided by height in meters squared; BMI data were primarily derived from the index pregnancy, but 17.6% were imputed from a later pregnancy and 0.04% came from diagnoses of obesity.

^b^ Percentages calculated of the total number of individuals with maternal diabetes in columns (complete cohort, n = 865; normotensive pregnancies, n = 739; HDP, n = 126).

**Figure 1.**
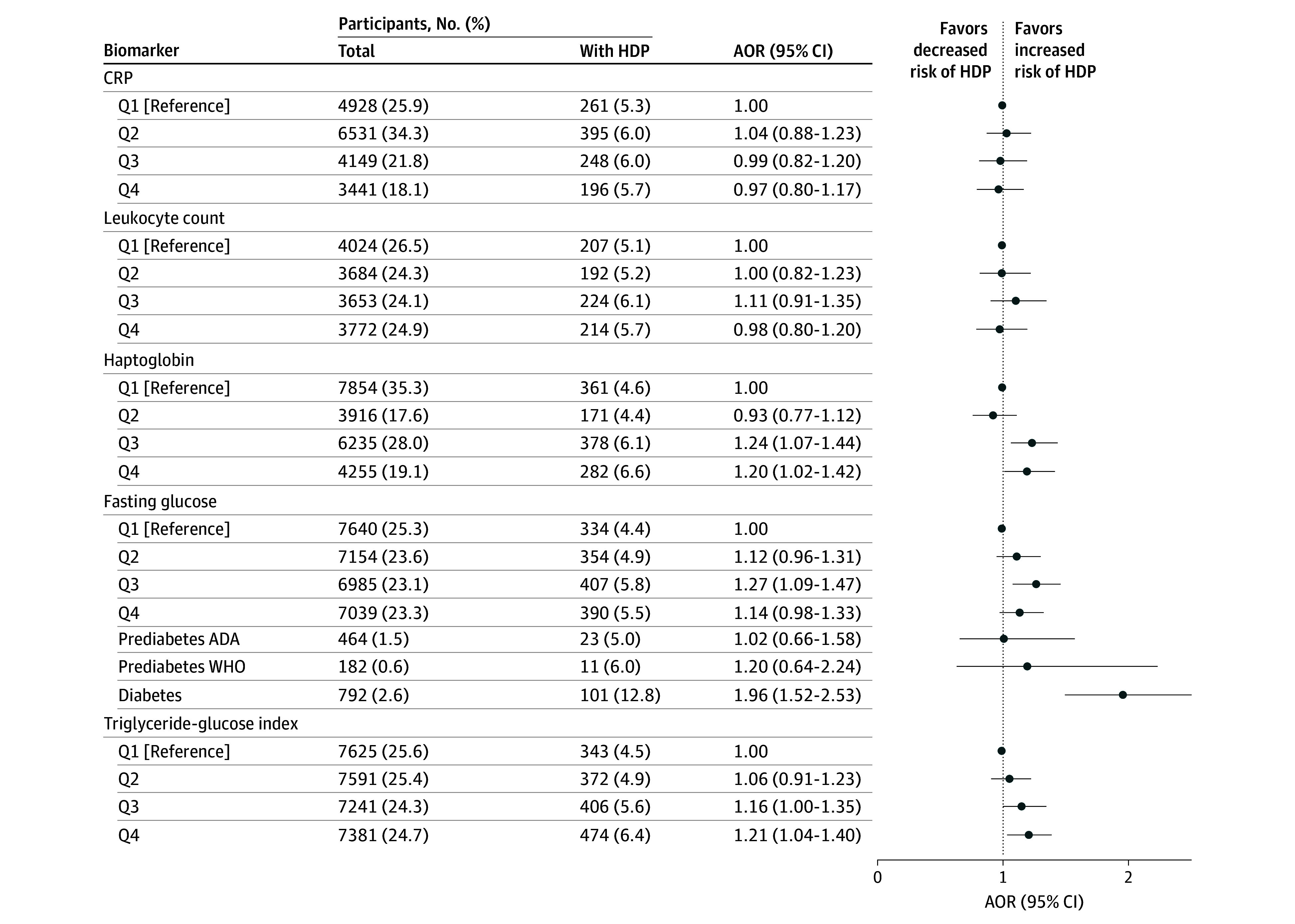
Dot Plots Showing Associations of Pregestational Inflammatory and Glucose Markers and Risk of Hypertensive Disorders of Pregnancy (HDP) in Nulliparous Women Biomarkers were analyzed as quartiles (Qs). Additional clinical cutoff categories for fasting glucose were prediabetes American Diabetes Association (ADA) of 100-125 mg/dL, prediabetes World Health Organization (WHO) of 110-125 mg/dL, and diabetes, of 126 mg/dL or higher, or known diabetes, the latter constituting 52.5% (to convert to mmol/L, multiply by 0.0555). Adjusted odds ratios (AORs) with 95% CIs were obtained from multivariable logistic regression analyses, after adjustments for body mass index in early pregnancy, maternal age at delivery, calendar year of pregnancy, pregestational chronic hypertension and dyslipidemia. In analyses of inflammatory markers, pregestational diabetes was also included in the adjusted model. Q1, Q2, Q3, and Q4 cutoffs were respectively ≤0.20, 0.21-0.40, 0.41-0.60, and >0.60 mg/dL for C-reactive protein (CRP; to convert to mg/L, multiply by 10); ≤5300, 5301-6300, 6301-7500, and >7500 /µL for leukocyte count (to convert to × 10^9^/L, multiply by 0.001); 80, 81-90, 91-110, and >110 mg/dL for haptoglobin (to convert to mg/L, multiply by 10); ≤74, 75-80, 81-85, and >85 mg/dL for fasting glucose (to convert to mmol/L, multiply by 0.0555); and ≤6.06, 6.07-6.37, 6.38-6.71, and >6.71 mg/dL for the triglyceride-glucose index.

**Figure 2.**
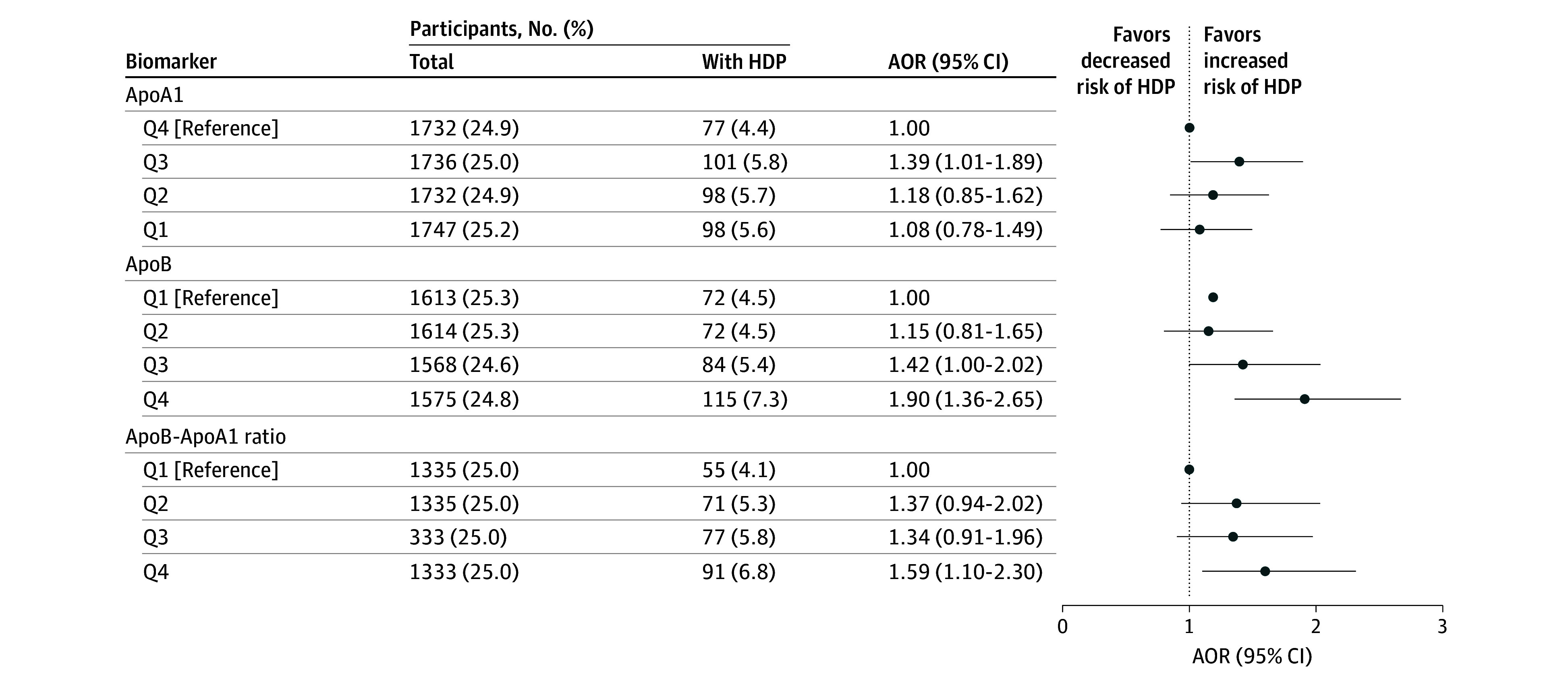
Dot Plots Showing Associations of Pregestational Apolipoproteins and Risk of Hypertensive Disorders of Pregnancy (HDP) in Nulliparous Women Adjusted odds ratios (AORs) with 95% CIs were obtained from multivariable logistic regression analyses, after adjustments for body mass index in early pregnancy, maternal age at delivery, calendar year of pregnancy, and pregestational chronic hypertension and diabetes. Quartile cutoffs (Q1, Q2, Q3, Q4) were respectively ≤132, 133-145, 146-160, and >160 mg/dL for apolipoprotein A1 (ApoA1); ≤77, 78-91, 92-106, and >106 mg/dL for apolipoprotein B (ApoB); and ≤0.51, 0.52-0.62, 0.63-0.75, and >0.75 for the ApoB/ApoA1 ratio for the ApoB/ApoA1 ratio (to convert ApoA1 and ApoB to g/L, multiply by 0.01).

**Figure 3.**
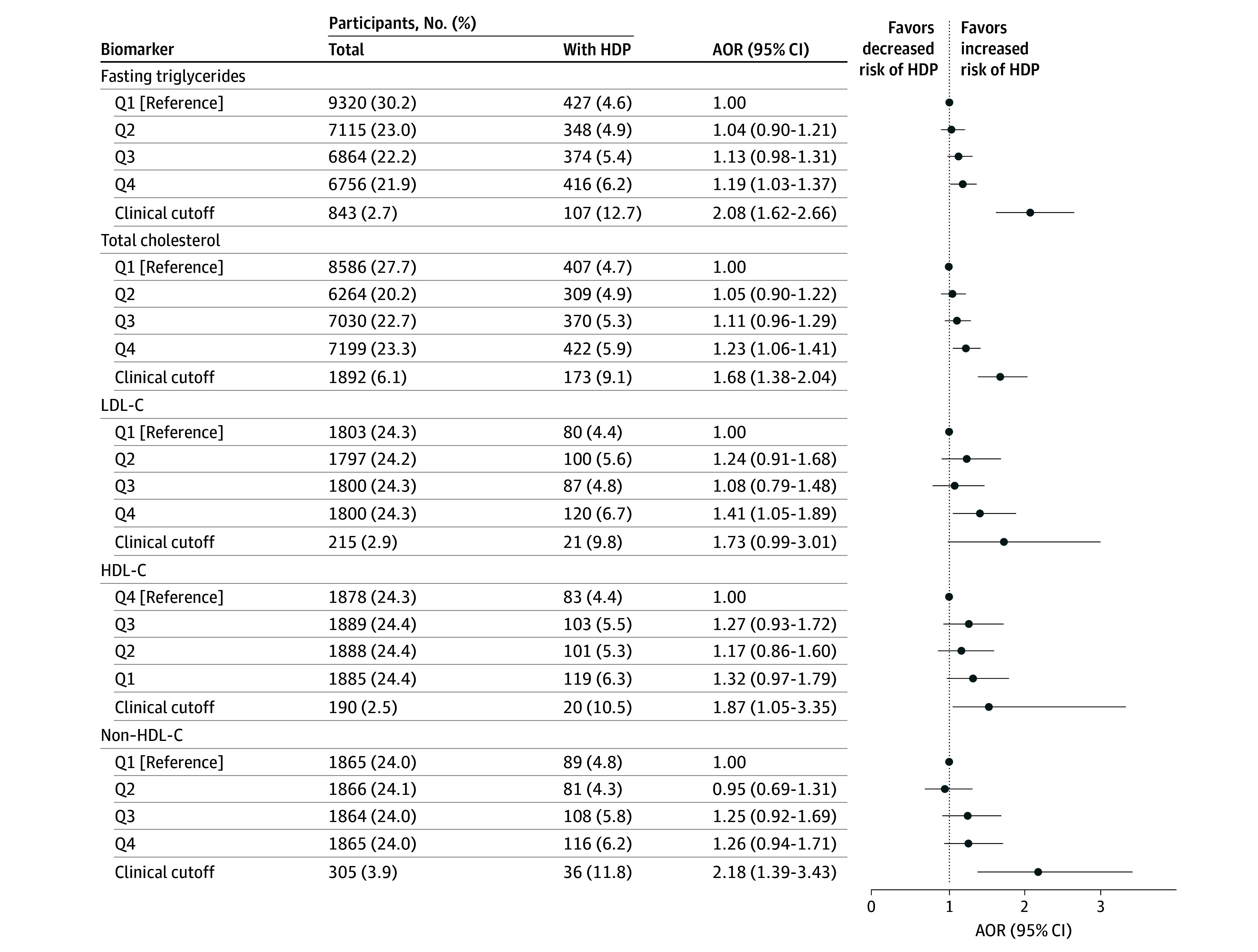
Dot Plots Showing Associations of Pregestational Clinically Used Lipid Markers and Risk of Hypertensive Disorders of Pregnancy (HDP) in Nulliparous Women Clinically used lipid markers were analyzed as quartiles (Qs) with an additional clinical category of clinical cutoff corresponding to diagnosed or biomarker-based dyslipidemia. Adjusted odds ratios (AORs) with 95% CIs were obtained from multivariable logistic regression analyses, after adjustments for body mass index in early pregnancy, maternal age at delivery, calendar year of pregnancy, and pregestational chronic hypertension and diabetes. Q1, Q2, Q3, and Q4 cutoffs were respectively ≤53, 54-71, 72-97, and >97 mg/dL for fasting triglycerides (to convert to mmol/L, multiply by 0.0113); ≤164, 165-179, 180-201, and >201 mg/dL for total cholesterol; ≤97, 98-115, 116-137, and >137 mg/dL for non–high-density lipoprotein cholesterol (non–HDL-C); ≤83, 84-100, 101-122, and >122 mg/dL for low-density lipoprotein cholesterol (LDL-C); and ≤56, 57-64, 65-75, and >75 mg/dL for HDL-C (to convert LDL-C, HDL-C, non–HDL-C, and total cholesterol to mmol/L, multiply by 0.0259).

Women with HDP had higher pregestational median levels of haptoglobin (SMD, 0.16), ApoB (SMD, 0.19), ApoB/ApoA1 ratio (SMD, 0.19), fasting triglycerides (SMD, 0.13), TC (SMD, 0.13), LDL-C (SMD, 0.18), non–HDL-C (SMD, 0.19), fasting glucose (SMD, 0.12), and TyG index (SMD, 0.14), and lower median HDL-C levels (SMD, −0.10) (Table 2 and eTable 5 in Supplement 1). The percentages in Q4 (Q1 for HDL-C) of women diagnosed with HDP varied between 5.5% (fasting glucose) and 7.3% (ApoB), whereas in the clinical categories it varied between 9.8% (LDL-C) and 12.8% (fasting glucose) (Figure 1, Figure 2, and Figure 3).

**Table 2.** Cardiometabolic Biomarker Distribution Comparing HDP and Normotensive Pregnancies

Exposure	Median (IQR)		SMD (normotensive vs HDP pregnancies)
	Normotensive pregnancies (n = 33 251)	HDP pregnancies (n = 1938)	
Inflammation			
CRP, mg/dL	0.40 (0.20-0.60)	0.40 (0.30-0.50)	0.02
Haptoglobin, mg/dL	90 (80-110)	100 (80-110)	0.16
Leukocyte count, /µL	6300 (5300-7500)	6400 (5400-7600)	0.06
Lipid metabolism			
ApoA1, mg/dL	150 (130-160)	140 (130-160)	−0.08
ApoB, mg/dL	90 (80-110)	100 (80-110)	0.19
ApoB/ApoA1 ratio	0.6 (0.5-0.7)	0.6 (0.5-0.8)	0.19
Fasting triglycerides, mg/dL	71 (53-97)	80 (53-106)	0.13
TC, mg/dL	182 (162-201)	186 (166-209)	0.13
LDL-C, mg/dL	101 (85-124)	104 (89-127)	0.18
HDL-C, mg/dL	66 (58-73)	62 (54-73)	−0.10
Non–HDL-C, mg/dL	116 (97-139)	124 (101-147)	0.19
Glucose metabolism			
Fasting glucose, mg/dL	81 (74-86)	81 (76-86)	0.12
TyG index	6.4 (6.1-6.7)	6.4 (6.1-6.8)	0.14

Abbreviations: ApoA1, apolipoprotein A1; ApoB, apolipoprotein B; CRP, C-reactive protein; HDL-C, high-density lipoprotein cholesterol; HDP, hypertensive disorders of pregnancy; LDL-C, low-density lipoprotein cholesterol; TC, total cholesterol; TyG, triglyceride-glucose index; SMD, standardized mean difference.

SI conversion factors: To convert ApoA1 and ApoB to g/L, multiply by 0.01; CRP to mg/L, multiply by 10; haptoglobin to mg/L, multiply by 10; LDL-C, HDL-C, non–HDL-C, and TC to mmol/L, multiply by 0.0259; triglycerides to mmol/L, multiply by 0.0113; and fasting glucose to mmol/L, multiply by 0.0555.

Elevated haptoglobin (≥91 mg/dL; to convert to mg/L, multiply by 10) was associated with increased HDP risk (Q3 adjusted OR [AOR], 1.24 [95% CI, 1.07-1.44]; Q4 AOR, 1.20 [95% CI, 1.02-1.42]). In contrast, CRP (Q4 AOR, 0.97 [95% CI, 0.80-1.17]) and leukocyte count (Q4 AOR, 0.98 [95% CI, 0.80-1.20]) were not associated with risk of HDP (Figure 1 and eTable 6 in Supplement 1).

All lipid markers were associated with increased risk of HDP at clinical cutoff levels, except LDL, which showed a 95% CI lower limit just below 1.00 (AOR, 1.73 [95% CI, 0.99-3.01]). Quartile analyses indicated increased HDP risk associated with Q4 of ApoB (AOR, 1.90 [95% CI, 1.36-2.65]), ApoB/ApoA1 ratio (AOR, 1.59 [95% CI, 1.10-2.30]), fasting triglycerides (AOR, 1.19 [95% CI, 1.03-1.37]), TC (AOR, 1.23 [95% CI, 1.06-1.41]), and LDL-C (AOR, 1.41 [95% CI, 1.05-1.89]), with a 95% CI lower limit close to 1.00 observed for Q3 of fasting triglycerides (AOR, 1.13 [95% CI, 0.98-1.31]) and TC (AOR, 1.11 [95% CI, 0.96-1.29]). No associations were found for either Q1 of HDL-C (AOR, 1.32 [95% CI, 0.97-1.79]) or ApoA1 (AOR, 1.08 [95% CI, 0.78-1.49]) (Figure 2 and 3 and eTable 6 in Supplement 1).

Diagnosed diabetes or fasting glucose of at least 126 mg/dL without a diagnosis of diabetes was associated with increased risk of HDP (AOR, 1.96 [95% CI, 1.52-2.53]), whereas no associations were observed for prediabetic categories (ADA AOR, 1.02 [95% CI, 0.66-1.58]; WHO AOR, 1.20 [95% CI, 0.64-2.24]) as well as Q3 (AOR, 1.27 [95% CI, 1.09-1.47]) and Q4 (AOR, 1.14 [95% CI, 0.98-1.33]) for fasting glucose. For Q4 of the TyG index, the AOR was 1.21 (95% CI, 1.04-1.40) (Figure 1 and eTable 6 in Supplement 1).

The results from sensitivity analyses (eTable 7-13 in Supplement 1) were similar to the main analyses, with some exceptions. Additional adjustments for smoking status yielded similar associations with HDP risk for clinical cutoffs for fasting triglycerides and TC with slightly higher risks (eTable 8 in Supplement 1). In each of the subgroup restriction analyses, clinically low levels of HDL-C showed increased risk of HDP (eTables 11-13 in Supplement 1). Q1 of ApoA1 was, unlike the main results, associated with increased HDP risk (eTable 11 in Supplement 1) in the analyses restricted to occupational health care referrals.

The graphs from the cubic splines modeling showed linear relationships between the biomarkers and the estimated probability of HDP when the tails of the biomarker distribution were not considered. The exceptions were HDL-C levels, where the probability of HDP was increased at both low and high values, and fasting glucose levels, for which a nonlinear association was observed (eFigure 2 in Supplement 1). Linear regression models showed approximately 20% increased odds of HDP for every unit increase in fasting triglycerides, TC, LDL-C, and non–HDL-C levels (eTable 14 in Supplement 1).

## Discussion

The principal findings of this study investigating the association between pregestational cardiometabolic disturbances and risk of HDP were that several markers of cardiometabolic dysregulation, assessed years (median, 4-6 [range, 0-31] years) before pregnancy, were associated with increased HDP risk in a first completed pregnancy. Although the absolute differences in rates of HDP were small, levels of fasting glucose and lipids outside clinical cutoffs for diagnoses were associated with increased HDP risk. For fasting triglycerides, TC, and LDL-C, associations were evident even at subclinical cutoff levels, suggesting that HDP risk may arise before reaching thresholds typically used when assessing HDP risk in the pregestational state. Additional associations were observed for elevated haptoglobin and ApoB levels, the ApoB/ApoA1 ratio, and the TyG index, whereas CRP levels and leukocyte count showed no associations. Although our main results did not identify an association between low levels of ApoA1 or HDL-C and HDP, our sensitivity analyses did. This study is among the largest^22,23^ to assess pregestational biomarkers in a population-based cohort of nulliparous women with prospectively evaluated HDP.

Our study extends previous knowledge on pregestational cardiometabolic risk profile in relation to HDP risk^21,22,23,24,25,26,41,42^ by showing associations already at subclinical levels. Direct comparisons with previous studies are limited, primarily due to differences in categorization of biomarker levels and inconsistent restriction based on parity. Nevertheless, among the biomarkers we addressed, there were consistencies for fasting triglyceride and non–HDL-C levels.^22,23,24,26,41,42^ The associations we observed between subclinical levels of fasting triglycerides, TC, and LDL-C and increased HDP risk are in line with previous reports showing elevated HDP risk associated with higher levels of triglycerides,^22,23,24,26,42^ TC,^23,41^ and LDL-C.^23,41^ For TC and LDL-C levels, however, some studies have also reported null associations.^25,26^

The observed association between diabetes and increased HDP risk aligns with current guidelines.^43^ In contrast, our findings of no association for prediabetes levels differ from Salman et al,^21^ who reported an association between impaired fasting glucose and mild preeclampsia in multiparous women, and from Retnakaran et al,^23^ who reported significant mean fasting glucose differences in nulliparous women without diabetes. However, neither of these studies adjusted for BMI. One of the few previous studies assessing inflammatory markers reported, consistent with our findings, no association between CRP and HDP risk, regardless of CRP sensitivity type.^24^ Hedderson et al^25^ identified associations for leukocyte count higher than 7.2 × 10^9^/L (corresponding to 7200 per µL), whereas our finding for count of 6310 to 7500 per µL in the crude analysis was attenuated after adjustments that included diabetes and dyslipidemia—covariates that were not adjusted for in the compared study.

Our results provide support for a previous suggestion of common antecedents of CVD and HDP.^44^ Possible pathophysiology behind the observed associations between pregestational cardiometabolic disturbances and increased HDP risk involve vascular-related biological mechanisms, which may contribute to a limited ability to adapt to pregnancy. In particular, endothelial dysfunction can lead to poor placental perfusion and organ involvement.^13,14^ A pregestational abnormal lipid profile may lead to atherogenic changes in maternal vasculature, which can interfere with trophoblast invasion and spiral artery remodeling, both critical for normal placental development.^14^ Our findings for the TyG index with regard to the HDP risk may reflect an altered vascular reactivity and oxidative stress caused by insulin resistance. The absence of similar associations for fasting glucose may relate to it reflecting a later stage of insulin resistance. Finally, the findings regarding haptoglobin and increased HDP risk suggest that chronic low-grade inflammation contributes to the development of HDP, possibly through vascular and placental inflammatory pathways.

Taken together with the existing literature, our findings support earlier clinical attention to cardiometabolic biomarkers, even at subclinical levels, in women of reproductive age. Practitioners may therefore consider incorporating these markers into preconceptional counseling, emphasizing lifestyle modification and structured follow-up; recent evidence supports positive effects of lifestyle interventions at subclinical biomarker levels.^45^ In an antenatal setting, these findings may contribute to further development of risk assessment tools and monitoring strategies.

### Strengths and Limitations

A key strength is the close alignment between mean age in our study cohort and contemporary global childbearing age patterns.^46^ The study also benefits from high-quality register data and a large population-based cohort. Several limitations should be noted. Despite comprehensive confounder adjustment, residual confounding remains possible. Chronic hypertension may be underadjusted due to limited blood pressure data. However, chronic hypertension is rare in this age group.^47^ Imputation of BMI from a later pregnancy may have led to a slight overestimation of BMI, although this shortcoming seems unlikely to materially affect the results. Given the number of biomarkers and the use of arbitrary quartile-based cutoff values for several biomarkers, and that no adjustment for multiple testing was made, the analyses are of an exploratory, hypothesis-generating nature. The generalizability of our results is limited to nulliparous women given the inclusion criteria. Although inclusion depended on blood samples obtained during a health care visit, most women were referred in connection with routine occupational health examinations offered by their workplace, mitigating concerns about overrepresentation of morbidity compared with the general population.

## Conclusions

This cohort study found that several cardiometabolic biomarkers assessed before pregnancy were associated with increased HDP risk, supporting the notion of shared antecedents with CVD. Associations observed at subclinical levels of the biomarker indicate that current thresholds for diagnoses may overlook individuals at risk, raising the question of whether HDP guidelines should be revisited. The study findings may open new avenues both in preconceptional counseling, in which a simple blood test could motivate lifestyle changes, and for refining risk assessment tools in antenatal settings. Future research should investigate whether preconceptional cardiometabolic biomarkers investigation and lifestyle interventions could reduce the risk of HDP and whether pharmacological interventions should be considered at subclinical levels for women of reproductive age.

## Notes

Supplement 1. **eTable 1.** Median time in years (and IQR) between biomarker sampling and index pregnancy, presented per biomarker **eTable 2.** National registers used for data in the study, types of data provided and availability **eTable 3.** Identification of diagnoses, including ICD and ATC codes used **eTable 4.** Availability of biomarker data **eTable 5.** Distribution of cardiometabolic biomarker levels by outcome groups (hyper- and normotensive pregnancies) **eTable 6.** Associations between pregestational cardiometabolic biomarkers and risk of HDP in nulliparous women **eTable 7.** Associations between pregestational cardiometabolic biomarkers and risk of HDP in nulliparous women with additional adjustments for polycystic ovarian syndrome **eTable 8.** Associations between pregestational cardiometabolic biomarkers and risk of HDP in nulliparous women with additional adjustments for smoking **eTable 9.** Associations between pregestational cardiometabolic biomarkers and risk of HDP in nulliparous women where BMI was handled as a continuous variable **eTable 10.** Associations between pregestational cardiometabolic biomarkers and risk of HDP after stratification by time between biomarker sampling and index pregnancy **eTable 11.** Associations between pregestational cardiometabolic biomarkers and risk of HDP when restricting to occupational healthcare referrals **eTable 12.** Associations between pregestational cardiometabolic biomarkers and risk of HDP when restricting to individuals with singleton pregnancies **eTable 13.** Associations between pregestational cardiometabolic biomarkers and risk of HDP when restricting to individuals with no prophylactic prenatal acetyl-salicylic acid use during index pregnancy **eFigure 1.** Flowchart of participant inclusion in the study **eFigure 2.** Predicted probability of HDP across the range of six selected biomarkers (panel A-F), modelled using restricted cubic splines with knots at the 5th, 27.5th, 50th, 72.5th and 95th percentiles **eTable 14.** Associations assessed through linear regression between pregestational cardiometabolic biomarkers and risk of HDP for biomarkers with a visual linearity in the restricted cubic splines models
eMethods.

Supplement 2.
Data Sharing Statement
