# Supplementary material for: Pregestational Cardiometabolic Biomarkers and Future Hypertensive Disorders of Pregnancy
Source: JAMA Netw Open. 2026 Apr 30;9(4):e2610037. doi: 10.1001/jamanetworkopen.2026.10037 (PMC13133693; doi:10.1001/jamanetworkopen.2026.10037)
Supplement: Supplement 1. — eTable 1. Median time in years (and IQR) between biomarker sampling and index pregnancy, presented per biomarker eTable 2. National registers used for data in the study, types of data provided and availability eTable 3. Identification of diagnoses, including ICD and ATC codes used eTable 4. Availability of biomarker data eTable 5. Distribution of cardiometabolic biomarker levels by outcome groups (hyper- and normotensive pregnancies) eTable 6. Associations between pregestational cardiometabolic biomarkers and risk of HDP in nulliparous women eTable 7. Associations between pregestational cardiometabolic biomarkers and risk of HDP in nulliparous women with additional adjustments for polycystic ovarian syndrome eTable 8. Associations between pregestational cardiometabolic biomarkers and risk of HDP in nulliparous women with additional adjustments for smoking eTable 9. Associations between pregestational cardiometabolic biomarkers and risk of HDP in nulliparous women where BMI was handled as a continuous variable eTable 10. Associations between pregestational cardiometabolic biomarkers and risk of HDP after stratification by time between biomarker sampling and index pregnancy eTable 11. Associations between pregestational cardiometabolic biomarkers and risk of HDP when restricting to occupational healthcare referrals eTable 12. Associations between pregestational cardiometabolic biomarkers and risk of HDP when restricting to individuals with singleton pregnancies eTable 13. Associations between pregestational cardiometabolic biomarkers and risk of HDP when restricting to individuals with no prophylactic prenatal acetyl-salicylic acid use during index pregnancy eFigure 1. Flowchart of participant inclusion in the study eFigure 2. Predicted probability of HDP across the range of six selected biomarkers (panel A-F), modelled using restricted cubic splines with knots at the 5th, 27.5th, 50th, 72.5th and 95th percentiles eTable 14. Associations assessed through linear regres [file jamanetwopen-e2610037-s001.pdf]

Supplementary Online Content

Qvick A, Sandström A, Norhammar A, et al. Pregestational cardiometabolic biomarkers and future hypertensive disorders of pregnancy. *JAMA Netw Open*. 2026;9(4):e2610037. doi:10.1001/jamanetworkopen.2026.10037

eTable 1. Median time in years (and IQR) between biomarker sampling and index pregnancy, presented per biomarker

eTable 2. National registers used for data in the study, types of data provided and availability

eTable 3. Identification of diagnoses, including ICD and ATC codes used

eTable 4. Availability of biomarker data

eTable 5. Distribution of cardiometabolic biomarker levels by outcome groups (hyper- and normotensive pregnancies)

eTable 6. Associations between pregestational cardiometabolic biomarkers and risk of HDP in nulliparous women

eTable 7. Associations between pregestational cardiometabolic biomarkers and risk of HDP in nulliparous women with additional adjustments for polycystic ovarian syndrome

eTable 8. Associations between pregestational cardiometabolic biomarkers and risk of HDP in nulliparous women with additional adjustments for smoking

eTable 9. Associations between pregestational cardiometabolic biomarkers and risk of HDP in nulliparous women where BMI was handled as a continuous variable

eTable 10. Associations between pregestational cardiometabolic biomarkers and risk of HDP after stratification by time between biomarker sampling and index pregnancy

eTable 11. Associations between pregestational cardiometabolic biomarkers and risk of HDP when restricting to occupational healthcare referrals

eTable 12. Associations between pregestational cardiometabolic biomarkers and risk of HDP when restricting to individuals with singleton pregnancies

eTable 13. Associations between pregestational cardiometabolic biomarkers and risk of HDP when restricting to individuals with no prophylactic prenatal acetyl-salicylic acid use during index pregnancy

eFigure 1. Flowchart of participant inclusion in the study

eFigure 2. Predicted probability of HDP across the range of six selected biomarkers (panel A-F), modelled using restricted cubic splines with knots at the 5th, 27.5th, 50th, 72.5th and 95th percentiles

eTable 14. Associations assessed through linear regression between pregestational cardiometabolic biomarkers and risk of HDP for biomarkers with a visual linearity in the restricted cubic splines models

eMethods.

eReferences.

This supplementary material has been provided by the authors to give readers additional information about their work.

© 2026 Qvick A et al. *JAMA Network Open*.

eTable 1. Median time in years (and IQR) between biomarker sampling and index pregnancy, presented per biomarker

	**Complete cohort**<br><br>n = 35 189<br>(100%)	**Normotensive pregnancies**<br><br>n = 33 251<br>(94.5 %)	**HDP pregnancies**<br><br>n = 1 938<br>(5.5 %)
**Inflammation**[a]			
CRP	4 (2-8)	4 (2-8)	6 (2-10)
Haptoglobin	4 (2-8)	4 (2-8)	5 (2-10)
Leukocyte count	6 (2-10)	5 (2-10)	7 (3-12)
**Lipid metabolism**[a]			
ApoA1	4 (2-8)	4 (2-8)	5 (2-9)
ApoB	4 (2-8)	4 (2-8)	6 (2-9)
ApoB/apoA1 ratio	4 (2-8)	4 (2-8)	5 (2-9)
Fasting triglycerides	4 (1-8)	4 (1-8)	5 (2-9)
TC	4 (1-8)	4 (1-8)	5 (2-9)
LDL-C	4 (2-8)	4 (2-8)	5 (2-9)
HDL-C	4 (2-8)	4 (2-8)	5 (2-9)
Non-HDL-C	4 (2-8)	4 (2-8)	5 (2-9)
**Glucose metabolism**[a]			
Fasting glucose	4 (1-8)	4 (1-8)	5 (2-9)
TyG index	4 (2-8)	4 (2-8)	5 (2-9)

ApoA1, apolipoprotein A-1; apoB, apolipoprotein B; CRP, C-reactive protein; HDL-C, high-density lipoprotein cholesterol; HDP, hypertensive disorders of pregnancy; IQR, interquartile range; LDL-C, low-density lipoprotein cholesterol; TC, total cholesterol; TyG, triglyceride glucose index.

[a] Biomarker-specific minimum and maximum time in years between biomarker sampling and index pregnancy: CRP, haptoglobin & leukocyte count, fasting triglycerides, TC, fasting glucose & TyG index 0-31; apoA1, apoB, apoB/apoA1 ratio, HDL-C, LDL-C & non-HDL-C 0-28.

© 2026 Qvick A et al. *JAMA Network Open*.

eTable 2. National registers used for data in the study, types of data provided and availability

Register	Type of data	Available from
Medical Birth Register	Data on approximately 98% of all births in Sweden (maternal and infant ante- & perinatal data)	1973-
National Patient Register, in-patients	Data on hospital admissions in Sweden by ICD-coded diagnoses	Regionally: 1964-<br>Nationally: 1987-
National Patient Register, out-patients	Data on out-patient visits in specialized health care by ICD-coded diagnoses	2001-
National Prescribed Drug Register	Data on prescribed and dispensed drugs in Sweden	July 2005-
Longitudinal integrated database for health insurance and labor market studies (LISA)	Sociodemographic data	1990-
National Diabetes Register	Data on types of diabetes and treatments from approximately 90% of all diabetes patients in primary care and specialized outpatient clinics	1996-

ICD, International Classification of Diseases.

© 2026 Qvick A et al. *JAMA Network Open*.

eTable 3. Identification of diagnoses, including ICD and ATC codes used

Diagnosis	Identification criteria	ICD-10-codes	ICD-9-codes	ICD-8-codes	ATC-codes
Gestational hypertension<br><br>(Part of the composite outcome HDP)	1) Received a diagnosis of chronic hypertension or gestational hypertension after 20 weeks of gestation until 1 week postpartum. The individual had not received any of these diagnoses before 20 weeks of gestation.<br><br>or<br><br>2) Dispatched prescribed antihypertensive drugs to treat gestational hypertension after 20 weeks of gestation until 1 week postpartum. Did not dispatch these drugs before 20 weeks of gestation.	**O13** Gestational hypertension<br>**O10** Pre-existing hypertension complicating pregnancy, childbirth, and the puerperium<br>**I10** Essentially (primary) hypertension<br>**I11** Hypertensive heart disease<br>**I12** Hypertensive renal disease<br>**I13** Hypertensive heart and renal disease<br>**I15** Secondary hypertension	**642.0** Benign essential hypertension complicating pregnancy, childbirth, and the puerperium<br>**642.1** Hypertension secondary to renal disease, complicating pregnancy, childbirth, and the puerperium<br>**642.2** Other pre-existing hypertension, complicating pregnancy, childbirth, and the puerperium<br>**642.9** Unspecified hypertension complicating pregnancy, childbirth, and the puerperium<br>**642.3** Transient hypertension of pregnancy<br>**401** Essential (primary) hypertension<br>**402** Hypertensive heart disease<br>**403** Hypertensive chronic kidney disease<br>**404** Hypertensive heart and chronic kidney disease<br>**405** Secondary hypertension	**400** Malignant hypertension<br>**401,99** Benign essential hypertension<br>**402,99** Essential hypertensive heart disease<br>**403,99** Essential hypertensive renal disease<br>**404,99** Essential hypertensive heart and renal disease	**C07AG01** Labetalol<br>**C07AB02** Metoprolol<br>**C08CA05** Nifedipin<br>**C07AA03** Pindolol<br>**C02DB02** Hydralazin
Preeclampsia superimposed on chronic hypertension<br><br>(Part of the composite outcome HDP)	1) Received a diagnosis of preeclampsia superimposed on chronic hypertension after 20 weeks of gestation.<br><br>or<br><br>2) Received a diagnosis of preeclampsia or eclampsia after 20 weeks of gestation and previously received a diagnosis of chronic or gestational hypertension before 20 weeks of gestation. The individual did not receive any hypertension diagnosis after 20 weeks of gestation.<br><br>or<br><br>3) Received a diagnosis of preeclampsia or eclampsia after 20 weeks of gestation and dispatched prescribed antihypertensive	1) **O11** Preeclampsia superimposed on chronic hypertension<br>2) **O13** Gestational hypertension<br>**O10** Pre-existing hypertension complicating pregnancy, childbirth and the puerperium<br>**I10** Essentially (primary) hypertension<br>**I11** Hypertensive heart disease<br>**I12** Hypertensive renal disease<br>**I13** Hypertensive heart and renal disease<br>**I15** Secondary hypertension<br><br>+<br><br>**O14** Preeclampsia<br>**O15** Eclampsia	1) **642.7** Preeclampsia or eclampsia with superimposed on pre-existing hypertension<br>2) **642.0** Benign essential hypertension complicating pregnancy, childbirth and the puerperium<br>**642.1** Hypertension secondary to renal disease, complicating pregnancy, childbirth and the puerperium<br>**642.2** Other pre-existing hypertension, complicating pregnancy, childbirth and the puerperium<br>**642.9** Unspecified hypertension complicating pregnancy, childbirth and the puerperium<br>**642.3** Transient hypertension during pregnancy, childbirth and the puerperium<br>**401** Essential (primary) hypertension<br>**402** Hypertensive heart disease<br>**403** Hypertensive chronic kidney disease<br>**404** Hypertensive heart and chronic kidney disease	2) **400** Malignant hypertension<br>**401,99** Benign essential hypertension<br>**402,99** Essential hypertensive heart disease<br>**403,99** Essential hypertensive renal disease<br>**404,99** Essential hypertensive heart and renal disease<br><br>+<br><br>**637** Preeclampsia, eclampsia and toxicosis	**C03A** Low-ceiling diuretics, thiazides<br>**C07** Beta blocking agents<br>**C08** Calcium channel blockers<br>**C09** Agents acting on the renin-angiotensin system

© 2026 Qvick A et al. *JAMA Network Open*.

eTable 3. Identification of diagnoses, including ICD and ATC codes used

	drugs to treat chronic hypertension before 20 weeks of gestation. The individual did not dispatch any of these drugs after 20 weeks of gestation.		**405** Secondary hypertension<br><br>+<br><br>**642.4** Mild or unspecified preeclampsia<br>**642.5** Severe preeclampsia<br>**642.6** Eclampsia		
Preeclampsia<br><br>(Part of the composite outcome HDP)	Received a diagnosis of preeclampsia or eclampsia after 20 weeks of gestation.	**O14** Preeclampsia<br>**O15** Eclampsia	**642.4** Mild or unspecified preeclampsia<br>**642.5** Severe preeclampsia<br>**642.6** Eclampsia	**637** Preeclampsia, eclampsia and toxicosis	-
Chronic hypertension	1) Received a diagnosis of chronic or gestational hypertension at any time before index pregnancy until 20 weeks of gestation. The individual had not received any of these diagnoses before 20 weeks of gestation.	**O13** Gestational hypertension<br>**O10** Pre-existing hypertension complicating pregnancy, childbirth and the puerperium<br>**I10** Essentially (primary) hypertension<br>**I11** Hypertensive heart disease<br>**I12** Hypertensive renal disease<br>**I13** Hypertensive heart and renal disease<br>**I15** Secondary hypertension	**642.0** Benign essential hypertension complicating pregnancy, childbirth, and the puerperium<br>**642.1** Hypertension secondary to renal disease, complicating pregnancy, childbirth, and the puerperium<br>**642.2** Other pre-existing hypertension, complicating pregnancy, childbirth, and the puerperium<br>**642.9** Unspecified hypertension complicating pregnancy, childbirth, and the puerperium<br>**642.3** Transient hypertension of pregnancy<br>**401** Essential (primary) hypertension<br>**402** Hypertensive heart disease<br>**403** Hypertensive chronic kidney disease<br>**404** Hypertensive heart and chronic kidney disease<br>**405** Secondary hypertension	**400** Malignant hypertension<br>**401,99** Benign essential hypertension<br>**402,99** Essential hypertensive heart disease<br>**403,99** Essential hypertensive renal disease<br>**404,99** Essential hypertensive heart and renal disease	-
Diabetes mellitus	1) Received a diagnosis of diabetes mellitus at any time before 20 weeks of gestation<br><br>or<br>.<br>2) Dispatched prescribed antidiabetic drugs at any time before 20 weeks of gestation.	**E10** Type 1 diabetes mellitus<br>**E11** Type 2 diabetes mellitus<br>**E12** Malnutrition-related diabetes mellitus<br>**E13** Other sepcified diabetes mellitus<br>**E14** Unspecified diabetes mellitus<br>**O24.0** Pre-existing type 1 diabetes mellitus<br>**O24.1** Pre-existing type 2 diabetes mellitus<br>**O24.2** Pre-existing malnutrition-related diabetes mellitus<br>**O24.3** Pre-existing	**250** Diabetes mellitus<br>**648.0** Diabetes mellitus complicating pregnancy, childbirth, or the puerperium	**250** Diabetes mellitus<br>**761,10** Other maternal conditions unrealted to pregnancy – Diabetes mellitus	**A10A** Insulins and analogues<br>**A10B** Blood glucose lowering drugs, excluding insulins<br>**A10X** Other drugs used in diabetes

eTable 3. Identification of diagnoses, including ICD and ATC codes used

		diabetes mellitus, unspecified			
Type 1 DM	1) Received a diagnosis of type 1 diabetes mellitus at any time before 20 weeks of gestation<br><br>or<br>.<br>2) Dispatched prescribed antidiabetic drugs (insulins alone) at any time before 20 weeks of gestation.	**E10** Type 1 diabetes mellitus<br>**O24.0** Pre-existing type 1 diabetes mellitus	-	-	**A10A** Insulins and analogues
Type 2 DM	1) Received a diagnosis of type 2 diabetes mellitus at any time before 20 weeks of gestation<br><br>or<br>.<br>2) Dispatched prescribed antidiabetic drugs (other drugs than insulins alone) at any time before 20 weeks of gestation.	**E11** Type 2 diabetes mellitus<br>**O24.1** Pre-existing type 2 diabetes mellitus	-	-	**A10A** Insulins and analogues<br>**A10B** Blood glucose lowering drugs, excluding insulins<br>**A10X** Other drugs used in diabetes
Other or unspecified DM	1) Received a diagnosis of other diabetes mellitus at any time before 20 weeks of gestation.	**E12** Malnutrition-related diabetes mellitus<br>**E13** Other specified diabetes mellitus<br>**E14** Unspecified diabetes mellitus<br>**O24.2** Pre-existing malnutrition-related diabetes mellitus<br>**O24.3** Pre-existing diabetes mellitus, unspecified	-	-	-
Systemic lupus erythematosus	Received a diagnosis of systemic lupus erythematosus at any time before 20 weeks of gestation.	**M32** Systemic lupus erythematosus	**710.0** Systemic lupus erythematosus	**734,10** Systemic lupus erythemathosus	-
Chronic kidney disease	Received a diagnosis of chronic kidney disease at any time before 20 weeks of gestation.	**N18** Chronic kidney disease	**585** Chronic kidney disease	**582** Chronic nephritis<br>**792,99** Casus male definiti - Uraemia	-
Dyslipidemia	1) Received a diagnosis of dyslipidemia at any time before 20 weeks of gestation<br><br>or<br><br>2) Dispatched any lipid-modifying agents at any time before 20 weeks of gestation.	**E78** Disorders of lipoprotein metabolism and other lipidaemias	**272** Disorders of lipid metabolism	**272** Congenital disorders of lipid metabolism<br>**279,00** Hypercholesterolaemia non familiaris sive NUD<br>**279,01** Hyperlipaemia, hyperglyceridaemia non familiaris NUD	**C10AA** HMG CoA reductase inhibitors<br>**C10AB** Fibrates<br>**C10AC** Bile acid sequestrants<br>**C10AD** Nicotonic acid and derivatives<br>**C10AX09** Ezetimibe<br>**C10B** Lipid modifying agents, combinations

© 2026 Qvick A et al. *JAMA Network Open*.

eTable 3. Identification of diagnoses, including ICD and ATC codes used

Gestational diabetes	Received a diagnosis of gestational diabetes at any time from pregnancy start to date of delivery.	**O24.4** Diabetes mellitus arising in pregnancy	**648.8** Abnormal glucose tolerance complicating pregnancy, childbirth, or the puerperium	-	-
Antiphospholipid syndrome	Received a diagnosis of antiphospholipid syndrome at any time before 20 weeks of gestation.	**D68.8** Other specified coagulation defects **D68.6A** Antiphospholipid-antibody syndrome	-	-	-
Cardiovascular disease	Received a diagnosis of any cardiovascular disease at any time before 20 weeks of gestation.	**I00-I02** Acute rheumatic fever **I05-I09** Chronic rheumatic heart disease **I10-I25** Ischemic heart diseases **I26-I28** Pulmonary heart disease and diseases of pulmonary circulation **I30-I52** Other forms of heart disease **I60-I69** Cerebrovascular diseases **I70-I79** Diseases of arteries, arterioles, and capillaries	**391** Acute rheumatic fever with heart disease **393–398** Chronic rheumatic heart diseases **410–417** Ischemic heart diseases **420-425** Valvular and pulmonary heart disease **426-427** Arrythmias **428-429** Heart failure and other forms of heart diseases **430-438** Cerebrovascular diseases **440-448** Diseases of arteries, arterioles, and capillaries	**390-392** Acute rheumatic fever **393-398** Chronic rheumatic heart disease **410-414** Ischemic heart disease **420-429** Other forms of heart disease **430-438** Cerebrovascular disease **440-448** Diseases of arteries, arterioles, and capillaries	-
Rheumatic heart disease	Received a diagnosis of rheumatic heart disease at any time before 20 weeks of gestation.	**I00-I02** Acute rheumatic fever **I05-I09** Chronic rheumatic heart disease	**391** Acute rheumatic fever with heart disease **393–398** Chronic rheumatic heart diseases	**390-392** Acute rheumatic fever **393-398** Chronic rheumatic heart disease	-
Ischemic heart disease	Received a diagnosis of ischemic heart disease at any time before 20 weeks of gestation.	**I10-I25** Ischemic heart diseases	**410–417** Ischemic heart diseases	**410-414** Ischemic heart disease	-
Pulmonary heart disease	Received a diagnosis of pulmonary heart disease at any time before 20 weeks of gestation.	**I26-I28** Pulmonary heart disease and diseases of pulmonary circulation	**420-425** Valvular and pulmonary heart disease	-	-
Other heart disease	Received a diagnosis of other heart disease at any time before 20 weeks of gestation.	**I30-I52** Other forms of heart disease	**426-427** Arrythmias **428-429** Heart failure and other forms of heart diseases	**420-429** Other forms of heart disease	-
Cerebrovascular disease	Received a diagnosis of cerebrovascular disease at any time before 20 weeks of gestation.	**I60-I69** Cerebrovascular diseases	**430-438** Cerebrovascular diseases	**430-438** Cerebrovascular disease	-
Artery disease	Received a diagnosis of artery disease at any time before 20 weeks of gestation.	**I70-I79** Diseases of arteries, arterioles, and capillaries	**440-448** Diseases of arteries, arterioles, and capillaries	**440-448** Diseases of arteries, arterioles, and capillaries	-
Obesity	BMI ≥30 kg/m^2 in early pregnancy or received a diagnosis	**E66** Overweight and obesity	**278.0** Overweight and obesity	**277** Obesity not specified as of endocrine origin	-

© 2026 Qvick A et al. *JAMA Network Open*.

eTable 3. Identification of diagnoses, including ICD and ATC codes used

	of obesity at any time before date of delivery.		**278.8** Other hyperalimentation	**278.9** Other and unspecified hyperalimentation	
Polycystic ovarian syndrome	Received a diagnosis of any polycystic ovarian syndrome at any time before 20 weeks of gestation.	**E28.1** Hypersecretion of ovarian androgens **E28.2** Polycystic ovarian syndrome **N97.0** Female infertility associated with anovulation	**256.1** Other ovarian hyperfunction **256.4** Polycystic ovaries **628.0** Infertility, female, associated with anovulation	**256.0** Ovarian dysfunction, hyperfunction **256.9** Ovarian dysfunction, other and unspecified **628** Sterility, female	

Data on ICD-codes were obtained from the National Patient Register for in- and out-patients, the Swedish Medical Birth Register, and the Swedish National Diabetes Register. ATC-code data were obtained from the National Prescribed Drug Register.

ATC, Anatomical Therapeutical; BMI, body mass index; ICD, International Classification of Diseases.

© 2026 Qvick A et al. *JAMA Network Open*.

eTable 4. Availability of biomarker data

Biomarker, n (%)	Complete cohort<br><br>n = 35 189<br>(100%)	Normotensive pregnancies<br><br>n = 33 251<br>(94.5 %)	HDP pregnancies<br><br>n = 1 938<br>(5.5 %)
**Inflammation**			
CRP	19 049 (54.1)	17 949 (54.0)	1100 (56.8)
Leukocyte count	15 133 (43.0)	14 296 (43.0)	837 (43.2)
Haptoglobin	22 270 (63.3)	21 078 (63.4)	1 192 (61.5)
**Lipid metabolism**			
ApoA1	6 610 (18.8)	6 260 (18.8)	350 (18.1)
ApoB	6 033 (17.1)	5 714 (17.2)	319 (16.5)
ApoB/ApoA1	5 000 (14.2)	4 730 (14.2)	270 (13.9)
Fasting triglycerides	30 584 (86.9)	28 932 (87.0)	1 652 (85.2)
TC	30 657 (87.1)	28 996 (87.2)	1 661 (85.7)
LDL-C	7 415 (21.1)	7 007 (21.1)	408 (21.1)
HDL-C	7 395 (21.0)	6 992 (21.0)	403 (20.8)
Non-HDL-C	7 429 (21.1)	7 022 (21.1)	407 (21.0)
**Glucose metabolism**			
Fasting glucose	29 934 (85.1)	28 335 (85.2)	1 599 (82.5)
TyG index	29 515 (83.9)	27 941 (84.0)	1 574 (81.2)

ApoA1, apolipoprotein A-1; apoB, apolipoprotein B; CRP, C-reactive protein; HDL-C, high-density lipoprotein cholesterol; HDP, hypertensive disorders of pregnancy; LDL-C, low-density lipoprotein cholesterol; TC, total cholesterol, TyG, triglyceride glucose.

© 2026 Qvick A et al. *JAMA Network Open*.

eTable 5. Distribution of cardiometabolic biomarker levels by outcome groups (hyper- and normotensive pregnancies)

Exposures	**Normotensive pregnancies** n (%)	**HDP pregnancies** n (%)
**CRP** (mg/dL)	*n = 17 949 (94.2 %)*	*n = 1 100 (5.8 %)*
Q1 (≤0.20)	4 667 (26.0)	261 (23.7)
Q2 (0.21-0.40)	6 136 (34.2)	395 (35.9)
Q3 (0.41-0.60)	3 901 (21.7)	248 (22.5)
Q4 (>0.60)	3 245 (18.1)	196 (17.8)
**Leukocyte count** (/µL)	*n = 14 296 (94.5 %)*	*n = 837 (5.5 %)*
Q1 (≤5300)	3 817 (26.7)	207 (24.7)
Q2 (5301-6300)	3 492 (24.4)	192 (22.9)
Q3 (6301-7500)	3 429 (24.0)	224 (26.8)
Q4 (>7500)	3 558 (24.9)	214 (25.6)
**Haptoglobin** (mg/dL)	*n = 21 078 (94.6 %)*	*n = 1 192 (5.4 %)*
Q1 (≤800)	7 493 (35.5)	361 (30.3)
Q2 (801-909)	3 745 (17.8)	171 (14.3)
Q3 (910-1100)	5 867 (27.8)	378 (31.7)
Q4 (>1100)	3 973 (18.8)	282 (23.7)
**ApoA1** (mg/dL)	*n = 6 573 (94.6 %)*	*n = 374 (5.4 %)*
Q4 (>160)	1 655 (25.2)	77 (20.6)
Q3 (146-160)	1 635 (24.9)	101 (27.0)
Q2 (133-145)	1 634 (24.9)	98 (26.2)
Q1 (≤132)	1 649 (25.1)	98 (26.2)
**ApoB** (mg/dL)	*n = 6 027 (94.6 %)*	*n = 343 (5.4 %)*
Q1 (≤77)	1 541 (25.6)	72 (21.0)
Q2 (78-91)	1 542 (25.6)	72 (21.0)
Q3 (92-106)	1 484 (24.6)	84 (24.5)
Q4 (>106)	1 460 (24.2)	115 (33.5)
**ApoB/ApoA1 ratio**	*n = 5 042 (94.5 %)*	*n = 294 (5.5 %)*
Q1 (≤0.51)	1 280 (25.4)	55 (18.7)
Q2 (0.52-0.62)	1 264 (25.1)	71 (24.1)
Q3 (0.63-0.75)	1 256 (24.9)	77 (26.2)
Q4 (>0.75)	1 242 (24.6)	91 (31.0)
**Fasting triglycerides** (mg/dL)	*n = 29 226 (94.6 %)*	*n = 1 672 (5.4 %)*
Q1 (≤53)	8 893 (30.4)	427 (25.5)
Q2 (54-71)	6 767 (23.1)	348 (20.8)
Q3 (72-97)	6 490 (22.2)	374 (22.4)
Q4 (>97)[a]	6 340 (21.7)	416 (24.9)
Dyslipidemia (elevated[b] or previous diagnosis)	736 (2.5)	107 (6.4)
**TC** (mg/dL)	*n = 29 290 (94.6 %)*	*n = 1 681 (5.4 %)*
Q1 (≤164)	8 179 (27.9)	407 (24.2)
Q2 (165-179)	5 955 (20.3)	309 (18.4)
Q3 (180-201)	6 660 (22.7)	370 (22.0)
Q4 (>201)[a]	6 777 (23.1)	422 (25.1)
Dyslipidemia (elevated[b] or previous diagnosis)	1 719 (5.9)	173 (10.3)
**LDL-C** (mg/dL)	*n = 7 007 (94.5 %)*	*n = 408 (5.5 %)*
Q1 (≤83)	1 723 (24.6)	80 (19.6)
Q2 (84-100)	1 697 (24.2)	100 (24.5)
Q3 (101-122)	1 713 (24.4)	87 (21.3)
Q4 (>122)[a]	1 680 (24.0)	120 (29.4)
Dyslipidemia (elevated[b] or previous diagnosis)	194 (2.8)	21 (5.1)
**HDL-C** (mg/dL)	*n = 7 304 (94.5 %)*	*n = 426 (5.5 %)*
Q4 (>75)	1 795 (24.6)	83 (19.5)
Q3 (65-75)	1 786 (24.5)	103 (24.2)
Q2 (57-64)	1 787 (24.5)	101 (23.7)
Q1 (≤56)[a]	1 766 (24.2)	119 (27.9)
Dyslipidemia (<39 or previous diagnosis)	170 (2.3)	20 (4.7)
**Non-HDL-C** (mg/dL)	*n = 7 335 (94.5 %)*	*n = 430 (5.5 %)*
Q1 (≤97)	1 776 (24.2)	89 (20.7)

© 2026 Qvick A et al. *JAMA Network Open.*

eTable 5. Distribution of cardiometabolic biomarker levels by outcome groups (hyper- and normotensive pregnancies)

Q2 (98-115)	1 785 (24.3)	81 (18.8)
Q3 (116-137)	1 756 (23.9)	108 (25.1)
Q4 (>137)[a]	1 749 (23.8)	116 (27.0)
Dyslipidemia (elevated[b] or previous diagnosis)	269 (3.7)	36 (8.4)
**Fasting glucose** (mg/dL)	*n = 28 636 (94.6 %)*	*n = 1 620 (5.4 %)*
Q1 (≤74)	7 306 (25.5)	334 (20.6)
Q2 (75-80)	6 800 (23.7)	354 (21.9)
Q3 (81-85)	6 578 (23.0)	407 (25.1)
Q4 (86-99)[a]	6 649 (23.2)	390 (24.1)
Prediabetic ADA (100-109)	441 (1.5)	23 (1.4)
Prediabetic WHO (110-125)	171 (0.6)	11 (0.7)
Diabetic (≥126 or previous diagnosis)	691 (2.4)	101 (6.2)
**TyG** index	*n = 28 243 (94.7 %)*	*n = 1 595 (5.3 %)*
Q1 (≤6.06)	7 282 (25.8)	343 (21.5)
Q2 (6.07-6.37)	7 219 (25.6)	372 (23.3)
Q3 (6.38-6.71)	6 835 (24.2)	406 (25.5)
Q4 (>6.71)	6 907 (24.5)	474 (29.7)

ApoA1, apolipoprotein A1; ApoB, apolipoprotein B; CRP, C-reactive protein; HDL-C, high-density lipoprotein cholesterol; HDP, hypertensive disorders of pregnancy; LDL-C, low-density lipoprotein cholesterol; Q, quartile; TC, total cholesterol; TyG, triglyceride-glucose.

[a] The quartiles were mutually exclusive with respect to the categories based on clinical cut-off values.

[b] Age-specific clinical cutoffs were used for fasting triglycerides (>248 mg/dL [1-17 years] and >230 mg/dL [≥18 years]), TC (>228 mg/dL [1-14 years], >232 mg/dL [15-17 years], >236 mg/dL [18-30 years], and >267 mg/dL [31-50 years]), LDL-C (>155 mg/dL [1-17 years], >166 mg/dL [18-30 years], >182 mg/dL [31-50 years]), and non-HDL-C (>201 mg/dL [<1 year], >166 mg/dL [1-9 years], >155 mg/dL [10-17 years], >182 mg/dL [18-29 years], >197 mg/dL [30-49 years], >240 mg/dL [>50 years])..

© 2026 Qvick A et al. *JAMA Network Open*.

eTable 6. Associations between pregestational cardiometabolic biomarkers and risk of HDP in nulliparous women.

Exposures	**Crude OR** (95 % CI)	**Adjusted[a] OR** (95 % CI)
**CRP** (mg/dL)<br>HDP, n = 1 100<br>Normotensive, n = 17 949		
Q1 (≤0.20)	1.00	1.00
Q2 (0.21-0.40)	1.15 (0.98-1.35)	1.04 (0.88-1.23)
Q3 (0.41-0.60)	1.14 (0.95-1.36)	0.99 (0.82-1.20)
Q4 (>0.60)	1.08 (0.89-1.31)	0.97 (0.80-1.17)
**Leukocyte count** (/µL)<br>HDP, n = 837<br>Normotensive, n = 14 296		
Q1 (≤5300)	1.00	1.00
Q2 (5301-6300)	1.01 (0.83-1.24)	1.00 (0.82-1.23)
Q3 (6301-7500)	1.20 (0.99-1.46)	1.11 (0.91-1.35)
Q4 (>7500)	1.11 (0.91-1.35)	0.98 (0.80-1.20)
**Haptoglobin** (mg/dL)<br>HDP, n = 1 192<br>Normotensive, n = 21 078		
Q1 (≤800)	1.00	1.00
Q2 (801-909)	0.95 (0.79-1.14)	0.93 (0.77-1.12)
Q3 (910-1100)	1.34 (1.15-1.55)	1.24 (1.07-1.44)
Q4 (>1100)	1.47 (1.26-1.73)	1.20 (1.02-1.42)
**ApoA1** (mg/dL)<br>HDP, n = 350<br>Normotensive, n = 6 260		
Q4 (>160)	1.00	1.00
Q3 (146-160)	1.41 (1.03-1.92)	1.39 (1.01-1.89)
Q2 (133-145)	1.23 (0.90-1.69)	1.18 (0.85-1.62)
Q1 (≤132)	1.26 (0.92-1.72)	1.08 (0.78-1.49)
**ApoB** (mg/dL)<br>HDP, n = 319<br>Normotensive, n = 5 714		
Q1 (≤77)	1.00	1.00
Q2 (78-91)	1.10 (0.77-1.56)	1.15 (0.81-1.65)
Q3 (92-106)	1.31 (0.93-1.84)	1.42 (1.00-2.02)
Q4 (>106)	1.85 (1.34-2.55)	1.90 (1.36-2.65)
**ApoB/ApoA1 ratio**<br>HDP, n = 270<br>Normotensive, n = 4 730		
Q1 (≤0.51)	1.00	1.00
Q2 (0.52-0.62)	1.30 (0.89-1.90)	1.37 (0.94-2.02)
Q3 (0.63-0.75)	1.39 (0.96-2.02)	1.34 (0.91-1.96)
Q4 (>0.75)	1.74 (1.22-2.49)	1.59 (1.10-2.30)
**Fasting triglycerides** (mg/dL)<br>HDP, n = 1 652<br>Normotensive, n = 28 932		
Q1 (≤53)	1.00	1.00
Q2 (54-71)	1.08 (0.93-1.25)	1.04 (0.90-1.21)
Q3 (72-97)	1.22 (1.05-1.40)	1.13 (0.98-1.31)
Q4 (>97)[b]	1.38 (1.20-1.58)	1.19 (1.03-1.37)
Dyslipidemia (elevated[c] or previous diagnosis)	3.06 (2.44-3.83)	2.08 (1.62-2.66)
**TC** (mg/dL)<br>HDP, n = 1 661<br>Normotensive, n = 28 996		
Q1 (≤164)	1.00	1.00
Q2 (165-179)	1.04 (0.89-1.21)	1.05 (0.90-1.22)
Q3 (180-201)	1.12 (0.97-1.30)	1.11 (0.96-1.29)
Q4 (>201)[b]	1.26 (1.09-1.45)	1.23 (1.06-1.41)
Dyslipidemia (elevated[c] or previous diagnosis)	2.02 (1.68-2.44)	1.68 (1.38-2.04)
**LDL-C** (mg/dL)<br>HDP, n = 408<br>Normotensive, n = 7 007		

© 2026 Qvick A et al. *JAMA Network Open.*

eTable 6. Associations between pregestational cardiometabolic biomarkers and risk of HDP in nulliparous women.

Q1 (≤83)	1.00	1.00
Q2 (84-100)	1.27 (0.94-1.72)	1.24 (0.91-1.68)
Q3 (101-122)	1.09 (0.80-1.49)	1.08 (0.79-1.48)
Q4 (>122)[b]	1.54 (1.15-2.06)	1.41 (1.05-1.89)
Dyslipidemia (elevated[c] or previous diagnosis)	2.33 (1.41-3.86)	1.73 (0.99-3.01)
**HDL-C** (mg/dL)<br>HDP, n = 426<br>Normotensive, n = 7 304		
Q4 (>75)	1.00	1.00
Q3 (65-75)	1.30 (0.96-1.76)	1.27 (0.93-1.72)
Q2 (57-64)	1.25 (0.92-1.70)	1.17 (0.86-1.60)
Q1 (≤56)[b]	1.52 (1.13-2.05)	1.32 (0.97-1.79)
Dyslipidemia (<39 or previous diagnosis)	2.58 (1.52-4.36)	1.87 (1.05-3.35)
**Non-HDL-C** (mgl/dL)<br>HDP, n = 430<br>Normotensive, n = 7 335		
Q1 (≤97)	1.00	1.00
Q2 (98-115)	0.98 (0.71-1.35)	0.95 (0.69-1.31)
Q3 (116-137)	1.32 (0.98-1.78)	1.25 (0.92-1.69)
Q4 (>137)[b]	1.41 (1.05-1.90)	1.26 (0.94-1.71)
Dyslipidemia (elevated[c] or previous diagnosis)	2.85 (1.88-4.33)	2.18 (1.39-3.43)
**Fasting glucose** (mg/dL)<br>HDP, n = 1 620<br>Normotensive, n = 28 636		
Q1 (≤74)	1.00	1.00
Q2 (75-80)	1.15 (0.99-1.34)	1.12 (0.96-1.31)
Q3 (81-85)	1.35 (1.16-1.57)	1.27 (1.09-1.47)
Q4 (86-99)[b]	1.29 (1.11-1.50)	1.14 (0.98-1.33)
Prediabetic ADA (100-109)	1.16 (0.75-1.79)	1.02 (0.66-1.58)
Prediabetic WHO (110-125)	1.42 (0.77-2.65)	1.20 (0.64-2.24)
Diabetic (≥126 or previous diagnosis)	3.18 (2.51-4.04)	1.96 (1.52-2.53)
**TyG index**<br>HDP, n = 1 595<br>Normotensive, n = 28 242		
Q1 (≤6.06)	1.00	1.00
Q2 (6.07-6.37)	1.11 (0.95-1.29)	1.06 (0.91-1.23)
Q3 (6.38-6.71)	1.28 (1.10-1.48)	1.16 (1.00-1.35)
Q4 (>6.71)	1.47 (1.27-1.70)	1.21 (1.04-1.40)

ApoA1, apolipoprotein A1; ApoB, apolipoprotein B; CI, confidence interval; CRP, C-reactive protein; HDL-C, high-density lipoprotein cholesterol; HDP, hypertensive disorders of pregnancy; LDL-C, low-density lipoprotein cholesterol; OR, odds ratio; Q, quartile; TC, total cholesterol; TyG, triglyceride-glucose.

[a] Adjusted for categorical body mass index in early pregnancy, maternal age at delivery, calendar year of pregnancy, and maternal chronic hypertension. In analyses of inflammatory and lipid markers, the model additionally included adjustment for maternal diabetes mellitus. In analyses of inflammatory and glucose markers, the model additionally included adjustment for maternal dyslipidemia.

[b] The quartiles were mutually exclusive with respect to the categories based on clinical cut-off values.

[c] Age-specific clinical cutoffs were used for fasting triglycerides (>248 mg/dL [1-17 years] and >230 mg/dL [≥18 years]), TC (>228 mg/dL [1-14 years], >232 mg/dL [15-17 years], >236 mg/dL [18-30 years], and >267 mg/dL [31-50 years]), LDL-C (>155 mg/dL [1-17 years], >166 mg/dL [18-30 years], >182 mg/dL [31-50 years]), and non-HDL-C (>201 mg/dL [<1 year], >166 mg/dL [1-9 years], >155 mg/dL [10-17 years], >182 mg/dL [18-29 years], >197 mg/dL [30-49 years], >240 mg/dL [>50 years])

eTable 7. Associations between pregestational cardiometabolic biomarkers and risk of HDP in nulliparous women with additional adjustments for polycystic ovarian syndrome

Exposures	**Crude OR** (95 % CI)	**Adjusted[a] OR** (95 % CI)
**CRP** (mg/dL)<br>HDP, n = 1 100<br>Normotensive, n = 17 949		
Q1 (≤0.20)	1.00	1.00
Q2 (0.21-0.40)	1.15 (0.98-1.35)	1.04 (0.88-1.23)
Q3 (0.41-0.60)	1.14 (0.95-1.36)	0.99 (0.83-1.20)
Q4 (>0.60)	1.08 (0.89-1.31)	0.96 (0.79-1.17)
**Leukocyte count** (/µL)<br>HDP, n = 837<br>Normotensive, n = 14 296		
Q1 (≤5300)	1.00	1.00
Q2 (5301-6300)	1.01 (0.83-1.24)	1.00 (0.82-1.23)
Q3 (6301-7500)	1.20 (0.99-1.46)	1.11 (0.91-1.35)
Q4 (>7500)	1.11 (0.91-1.35)	0.98 (0.80-1.20)
**Haptoglobin** (mg/dL)<br>HDP, n = 1 192<br>Normotensive, n = 21 078		
Q1 (≤800)	1.00	1.00
Q2 (801-909)	0.95 (0.79-1.14)	0.93 (0.77-1.12)
Q3 (910-1100)	1.34 (1.15-1.55)	1.24 (1.07-1.45)
Q4 (>1100)	1.47 (1.26-1.73)	1.21 (1.02-1.43)
**ApoA1** (mg/dL)<br>HDP, n = 374<br>Normotensive, n = 6 573		
Q4 (>160)	1.00	1.00
Q3 (146-160)	1.41 (1.03-1.92)	1.39 (1.02-1.90)
Q2 (133-145)	1.23 (0.90-1.69)	1.17 (0.85-1.61)
Q1 (≤132)	1.26 (0.92-1.72)	1.08 (0.78-1.49)
**ApoB** (mg/dL)<br>HDP, n = 343<br>Normotensive, n = 6 027		
Q1 (≤77)	1.00	1.00
Q2 (78-91)	1.10 (0.77-1.56)	1.16 (0.81-1.65)
Q3 (92-106)	1.31 (0.93-1.84)	1.42 (1.00-2.01)
Q4 (>106)	1.85 (1.34-2.55)	1.89 (1.35-2.63)
**ApoB/ApoA1 ratio**<br>HDP, n = 294<br>Normotensive, n = 5042		
Q1 (≤0.51)	1.00	1.00
Q2 (0.52-0.62)	1.30 (0.89-1.90)	1.37 (0.93-2.01)
Q3 (0.63-0.75)	1.39 (0.96-2.02)	1.33 (0.91-1.95)
Q4 (>0.75)	1.74 (1.22-2.49)	1.58 (1.09-2.28)
**Fasting triglycerides** (mg/dL)<br>HDP, n = 1 672<br>Normotensive, n = 29 226		
Q1 (≤53)	1.00	1.00
Q2 (54-71)	1.08 (0.93-1.25)	1.04 (0.90-1.21)
Q3 (72-97)	1.22 (1.05-1.40)	1.13 (0.98-1.31)
Q4 (>97)[b]	1.38 (1.20-1.58)	1.19 (1.03-1.37)
Dyslipidemia (elevated[c] or previous diagnosis)	3.06 (2.44-3.83)	2.07 (1.62-2.66)
**TC** (mgl/dL)<br>HDP, n = 1 681<br>Normotensive, n = 29 290		
Q1 (≤164)	1.00	1.00
Q2 (165-179)	1.04 (0.89-1.21)	1.05 (0.90-1.22)
Q3 (180-201)	1.12 (0.97-1.30)	1.11 (0.96-1.29)
Q4 (>201)[b]	1.26 (1.09-1.45)	1.23 (1.06-1.42)
Dyslipidemia (elevated[c] or previous diagnosis)	2.02 (1.68-2.44)	1.68 (1.38-2.04)
**LDL-C** (mg/dL)<br>HDP, n = 480<br>Normotensive, n = 7 007		

© 2026 Qvick A et al. *JAMA Network Open*.

eTable 7. Associations between pregestational cardiometabolic biomarkers and risk of HDP in nulliparous women with additional adjustments for polycystic ovarian syndrome

Q1 (≤83)	1.00	1.00
Q2 (84-100)	1.27 (0.94-1.72)	1.23 (0.91-1.67)
Q3 (101-122)	1.09 (0.80-1.49)	1.08 (0.79-1.48)
Q4 (>122)[b]	1.54 (1.15-2.06)	1.41 (1.05-1.89)
Dyslipidemia (elevated[c] or previous diagnosis)	2.33 (1.41-3.86)	1.73 (0.99-3.02)
**HDL-C** (mg/dL)<br>HDP, n = 426<br>Normotensive, n = 7 304		
Q4 (>75)	1.00	1.00
Q3 (65-75)	1.30 (0.95-1.76)	1.27 (0.93-1.73)
Q2 (57-64)	1.25 (0.92-1.70)	1.17 (0.86-1.60)
Q1 (≤56)[b]	1.52 (1.13-2.05)	1.31 (0.97-1.79)
Dyslipidemia (<39 or previous diagnosis)	2.58 (1.52-4.36)	1.90 (1.06-3.40)
**Non-HDL-C** (mg/dL)<br>HDP, n = 430<br>Normotensive, n = 7 335		
Q1 (≤97)	1.00	1.00
Q2 (98-115)	0.98 (0.71-1.35)	0.95 (0.69-1.31)
Q3 (116-137)	1.32 (0.98-1.78)	1.25 (0.92-1.69)
Q4 (>137)[b]	1.41 (1.05-1.90)	1.26 (0.94-1.71)
Dyslipidemia (elevated[c] or previous diagnosis)	2.85 (1.88-4.33)	2.17 (1.38-3.41)
**Fasting glucose** (mg/dL)<br>HDP, n = 1 620<br>Normotensive, n = 28 636		
Q1 (≤74)	1.00	1.00
Q2 (75-80)	1.15 (0.99-1.34)	1.12 (0.96-1.31)
Q3 (81-85)	1.35 (1.16-1.57)	1.27 (1.09-1.47)
Q4 (86-99)[b]	1.29 (1.11-1.50)	1.14 (0.98-1.33)
Prediabetic ADA (100-109)	1.16 (0.75-1.79)	1.02 (0.66-1.58)
Prediabetic WHO (110-125)	1.42 (0.77-2.65)	1.20 (0.64-2.24)
Diabetic (≥126 or previous diagnosis)	3.18 (2.51-4.04)	1.95 (1.51-2.52)
**TyG** index<br>HDP, n = 1 595<br>Normotensive, n = 28 242		
Q1 (≤6.06)	1.00	1.00
Q2 (6.07-6.37)	1.11 (0.95-1.29)	1.06 (0.91-1.23)
Q3 (6.38-6.71)	1.28 (1.10-1.48)	1.16 (1.00-1.35)
Q4 (>6.71)	1.47 (1.27-1.70)	1.21 (1.04-1.40)

ApoA1, apolipoprotein A1; ApoB, apolipoprotein B; CI, confidence interval; CRP, C-reactive protein; HDL-C, high-density lipoprotein cholesterol; HDP, hypertensive disorders of pregnancy; LDL, low-density lipoprotein cholesterol-C; OR, odds ratio; Q, quartile; TC, total cholesterol; TyG, triglyceride-glucose.

[a] Adjusted for categorical body mass index in early pregnancy, maternal age at delivery, calendar year of pregnancy, maternal chronic hypertension, and polycystic ovarian syndrome. In analyses of inflammatory and lipid markers, the model additionally included adjustment for maternal diabetes mellitus. In analyses of inflammatory and glucose markers, the model additionally included adjustment for maternal dyslipidemia.

[b] The quartiles were mutually exclusive with respect to the categories based on clinical cut-off values.

[c] Age-specific clinical cutoffs were used for fasting triglycerides (>248 mg/dL [1-17 years] and >230 mg/dL [≥18 years]), TC (>228 mg/dL [1-14 years], >232 mg/dL [15-17 years], >236 mg/dL [18-30 years], and >267 mg/dL [31-50 years]), LDL-C (>155 mg/dL [1-17 years], >166 mg/dL [18-30 years], >182 mg/dL [31-50 years]), and non-HDL-C (>201 mg/dL [<1 year], >166 mg/dL [1-9 years], >155 mg/dL [10-17 years], >182 mg/dL [18-29 years], >197 mg/dL [30-49 years], >240 mg/dL [>50 years]).

© 2026 Qvick A et al. *JAMA Network Open.*

eTable 8. Associations between pregestational cardiometabolic biomarkers and risk of HDP in nulliparous women with additional adjustments for smoking

Exposures	**Crude OR** (95 % CI)	**Adjusted[a] OR** (95 % CI)
**CRP** (mg/dL)<br>HDP, n = 1 032<br>Normotensive, n = 16 960		
Q1 (≤0.20)	1.00	1.00
Q2 (0.21-0.40)	1.15 (0.98-1.35)	1.00 (0.84-1.18)
Q3 (0.41-0.60)	1.14 (0.95-1.36)	0.96 (0.79-1.16)
Q4 (>0.60)	1.08 (0.89-1.31)	0.96 (0.78-1.16)
**Leukocyte count** (/µL)<br>HDP, n = 779<br>Normotensive, n = 13 484		
Q1 (≤5300)	1.00	1.00
Q2 (5301-6300)	1.01 (0.83-1.24)	1.03 (0.83-1.27)
Q3 (6301-7500)	1.20 (0.99-1.46)	1.16 (0.95-1.43)
Q4 (>7500)	1.11 (0.91-1.35)	1.02 (0.83-1.26)
**Haptoglobin** (mg/dL)<br>HDP, n = 1 125<br>Normotensive, n = 19 955		
Q1 (≤800)	1.00	1.00
Q2 (801-909)	0.95 (0.79-1.14)	0.92 (0.76-1.12)
Q3 (910-1100)	1.34 (1.15-1.55)	1.24 (1.06-1.45)
Q4 (>1100)	1.47 (1.26-1.73)	1.24 (1.05-1.48)
**ApoA1** (mg/dL)<br>HDP, n = 332<br>Normotensive, n = 5 925		
Q4 (>160)	1.00	1.00
Q3 (146-160)	1.41 (1.03-1.92)	1.35 (0.98–1.86)
Q2 (133-145)	1.23 (0.90-1.69)	1.14 (0.82-1.58)
Q1 (≤132)	1.26 (0.92-1.72)	1.04 (0.75-1.45)
**ApoB** (mg/dL)<br>HDP, n = 298<br>Normotensive, n = 5 410		
Q1 (≤77)	1.00	1.00
Q2 (78-91)	1.10 (0.77-1.56)	1.09 (0.75-1.58)
Q3 (92-106)	1.31 (0.93–1.84)	1.31 (0.91-1.89)
Q4 (>106)	1.85 (1.34-2.55)	1.95 (1.39-2.74)
**ApoB/ApoA1 ratio**<br>HDP, n = 270<br>Normotensive, n = 4 725		
Q1 (≤0.51)	1.00	1.00
Q2 (0.52-0.62)	1.30 (0.89-1.90)	1.29 (0.87-1.90)
Q3 (0.63-0.75)	1.39 (0.96-2.02)	1.16 (0.79-1.72)
Q4 (>0.75)	1.74 (1.22-2.49)	1.50 (1.03-2.19)
**Fasting triglycerides** (mg/dL)<br>HDP, n = 1 649<br>Normotensive, n = 28 922		
Q1 (≤53)	1.00	1.00
Q2 (54-71)	1.08 (0.93-1.25)	1.09 (0.94-1.27)
Q3 (72-97)	1.22 (1.05-1.40)	1.11 (0.96-1.30)
Q4 (>97)[b]	1.38 (1.20-1.58)	1.23 (1.06-1.43)
Dyslipidemia (elevated[c] or previous diagnosis)	3.06 (2.44-3.83)	2.22 (1.72-2.87)
**TC** (mg/dL)<br>HDP, n = 1 557<br>Normotensive, n = 27 371		
Q1 (≤164)	1.00	1.00
Q2 (165-179)	1.04 (0.89-1.21)	1.08 (0.92-1.26)
Q3 (180-201)	1.12 (0.97-1.30)	1.13 (0.97-1.31)
Q4 (>201)[b]	1.26 (1.09-1.45)	1.23 (1.06-1.42)
Dyslipidemia (elevated[c] or previous diagnosis)	2.02 (1.68-2.44)	1.77 (1.44-2.16)
**LDL-C** (mg/dL)<br>HDP, n = 387<br>Normotensive, n = 6 627		

eTable 8. Associations between pregestational cardiometabolic biomarkers and risk of HDP in nulliparous women with additional adjustments for smoking

Q1 (≤983)	1.00	1.00
Q2 (84-100)	1.27 (0.94-1.72)	1.22 (0.89-1.66)
Q3 (101-122)	1.09 (0.80-1.49)	1.05 (0.76-1.44)
Q4 (>122)[b]	1.54 (1.15-2.06)	1.37 (1.02-1.86)
Dyslipidemia (elevated[c] or previous diagnosis)	2.33 (1.41-3.86)	1.66 (0.93-2.96)
**HDL-C** (mg/dL)<br>HDP, n = 383<br>Normotensive, n = 6 611		
Q4 (>75)	1.00	1.00
Q3 (65-75)	1.30 (0.95-1.76)	1.21 (0.89-1.66)
Q2 (57-64)	1.25 (0.92-1.70)	1.12 (0.81-1.54)
Q1 (≤56)[b]	1.52 (1.13-2.05)	1.27 (0.93-1.74)
Dyslipidemia (<39 or previous diagnosis)	2.58 (1.52-4.36)	1.77 (0.96-3.24)
**Non-HDL-C** (mg/dL)<br>HDP, n = 387<br>Normotensive, n = 6 641		
Q1 (≤97)	1.00	1.00
Q2 (98-115)	0.98 (0.71-1.35)	0.94 (0.68-1.31)
Q3 (116-137)	1.32 (0.98-1.78)	1.19 (0.88-1.62)
Q4 (>137)[b]	1.41 (1.05-1.90)	1.24 (0.92-1.69)
Dyslipidemia (elevated[c] or previous diagnosis)	2.85 (1.88-4.33)	2.18 (1.37-3.49)
**Fasting glucose** (mg/dL)<br>HDP, n = 1 498<br>Normotensive, n = 26 759		
Q1 (≤74)	1.00	1.00
Q2 (75-80)	1.15 (0.99-1.34)	1.13 (0.96-1.33)
Q3 (81-85)	1.35 (1.16-1.57)	1.27 (1.09-1.49)
Q4 (86-99)[b]	1.29 (1.11-1.50)	1.14 (0.97-1.34)
Prediabetic ADA (100-109)	1.16 (0.75-1.79)	1.05 (0.67-1.64)
Prediabetic WHO (110-125)	1.42 (0.77-2.65)	1.32 (0.70-2.47)
Diabetic (≥126 or previous diagnosis)	3.18 (2.51-4.04)	1.96 (1.50-2.56)
**TyG index**<br>HDP, n = 1 474<br>Normotensive, n = 26 380		
Q1 (≤6.06)	1.00	1.00
Q2 (6.07-6.37)	1.11 (0.95-1.29)	1.10 (0.94-1.29)
Q3 (6.38-6.71)	1.28 (1.10-1.48)	1.16 (0.99-1.36)
Q4 (>6.71)	1.47 (1.27-1.70)	1.26 (1.08-1.47)

ApoA1, apolipoprotein A1; ApoB, apolipoprotein B; CI, confidence interval; CRP, C-reactive protein; HDL-C, high-density lipoprotein cholesterol; HDP, hypertensive disorders of pregnancy; LDL-C, low-density lipoprotein cholesterol; OR, odds ratio; Q, quartile; TC, total cholesterol; TyG, triglyceride-glucose.

[a] Adjusted for categorical body mass index in early pregnancy, maternal age at delivery, calendar year of pregnancy, maternal chronic hypertension, and smoking. In analyses of inflammatory and lipid markers, the model additionally included adjustment for maternal diabetes mellitus. In analyses of inflammatory and glucose markers, the model additionally included adjustment for maternal dyslipidemia.

[b] The quartiles were mutually exclusive with respect to the categories based on clinical cut-off values.

[c] Age-specific clinical cutoffs were used for fasting triglycerides (>248 mg/dL [1-17 years] and >230 mg/dL [≥18 years]), TC (>228 mg/dL [1-14 years], >232 mg/dL [15-17 years], >236 mg/dL [18-30 years], and >267 mg/dL [31-50 years]), LDL-C (>155 mg/dL [1-17 years], >166 mg/dL [18-30 years], >182 mg/dL [31-50 years]), and non-HDL-C (>201 mg/dL [<1 year], >166 mg/dL [1-9 years], >155 mg/dL [10-17 years], >182 mg/dL [18-29 years], >197 mg/dL [30-49 years], >240 mg/dL [>50 years]).

© 2026 Qvick A et al. *JAMA Network Open*.

eTable 9. Associations between pregestational cardiometabolic biomarkers and risk of HDP in nulliparous women where BMI was handled as a continuous variable

Exposures	**Crude OR** (95 % CI)	**Adjusted[a] OR** (95 % CI)
**CRP** (mg/dL)<br>HDP, n = 1 098<br>Normotensive, n = 17 943		
Q1 (≤0.20)	1.00	1.00
Q2 (0.21-0.40)	1.15 (0.98-1.35)	1.04 (0.88-1.24)
Q3 (0.41-0.60)	1.14 (0.95-1.36)	0.99 (0.82-1.19)
Q4 (>0.60)	1.08 (0.89-1.31)	0.95 (0.78-1.16)
**Leukocyte count** (/µL)<br>HDP, n = 836<br>Normotensive, n = 14 292		
Q1 (≤5300)	1.00	1.00
Q2 (5301-6300)	1.01 (0.83-1.24)	0.99 (0.81-1.22)
Q3 (6301-7500)	1.20 (0.99-1.46)	1.10 (0.90-1.34)
Q4 (>7500)	1.11 (0.91-1.35)	0.98 (0.80-1.19)
**Haptoglobin** (mg/dL)<br>HDP, n = 1 190<br>Normotensive, n = 21 072		
Q1 (≤800)	1.00	1.00
Q2 (801-909)	0.95 (0.79-1.14)	0.92 (0.77-1.11)
Q3 (910-1100)	1.34 (1.15-1.55)	1.22 (1.05-1.42)
Q4 (>1100)	1.47 (1.26-1.73)	1.18 (1.00-1.40)
**ApoA1** (mg/dL)<br>HDP, n = 350<br>Normotensive, n = 6 255		
Q4 (>160)	1.00	1.00
Q3 (146-160)	1.41 (1.03-1.92)	1.38 (1.01-1.89)
Q2 (133-145)	1.23 (0.90-1.69)	1.17 (0.85-1.61)
Q1 (≤132)	1.26 (0.92-1.72)	1.07 (0.77-1.48)
**ApoB** (mg/dL)<br>HDP, n = 319<br>Normotensive, n = 5 709		
Q1 (≤77)	1.00	1.00
Q2 (78-91)	1.10 (0.77-1.56)	1.15 (0.80-1.65)
Q3 (92-106)	1.31 (0.93-1.84)	1.42 (1.00-2.02)
Q4 (>106)	1.85 (1.34-2.55)	1.93 (1.38-2.69)
**ApoB/ApoA1 ratio**<br>HDP, n = 294<br>Normotensive, n = 5042		
Q1 (≤0.51)	1.00	1.00
Q2 (0.52-0.62)	1.30 (0.89-1.90)	1.36 (0.93-2.00)
Q3 (0.63-0.75)	1.39 (0.96-2.02)	1.36 (0.93-1.99)
Q4 (>0.75)	1.74 (1.22-2.49)	1.62 (1.12-2.34)
**Fasting triglycerides** (mg/dL)<br>HDP, n = 1 672<br>Normotensive, n = 29 226		
Q1 (≤53)	1.00	1.00
Q2 (54-71)	1.08 (0.93-1.25)	1.04 (0.90-1.20)
Q3 (72-97)	1.22 (1.05-1.40)	1.12 (0.97-1.30)
Q4 (>97)[b]	1.38 (1.20-1.58)	1.18 (1.02-1.36)
Dyslipidemia (elevated[c] or previous diagnosis)	3.06 (2.44-3.83)	2.04 (1.59-2.62)
**TC** (mg/dL)<br>HDP, n = 1 658<br>Normotensive, n = 28 986		
Q1 (≤164)	1.00	1.00
Q2 (165-179)	1.04 (0.89-1.21)	1.03 (0.89-1.21)
Q3 (180-201)	1.12 (0.97-1.30)	1.10 (0.95-1.28)
Q4 (>201)[b]	1.26 (1.09-1.45)	1.21 (1.05-1.40)
Dyslipidemia (elevated[c] or previous diagnosis)	2.02 (1.68-2.44)	1.66 (1.37-2.02)
**LDL-C** (mg/dL)<br>HDP, n = 407<br>Normotensive, n = 7 002		

© 2026 Qvick A et al. *JAMA Network Open*.

eTable 9. Associations between pregestational cardiometabolic biomarkers and risk of HDP in nulliparous women where BMI was handled as a continuous variable

Q1 (≤83)	1.00	1.00
Q2 (84-100)	1.27 (0.94-1.72)	1.22 (0.90-1.66)
Q3 (101-122)	1.09 (0.80-1.49)	1.08 (0.79-1.48)
Q4 (>122)[b]	1.54 (1.15-2.06)	1.42 (1.06-1.91)
Dyslipidemia (elevated[c] or previous diagnosis)	2.33 (1.41-3.86)	1.72 (0.99-3.01)
**HDL-C** (mg/dL)<br>HDP, n = 402<br>Normotensive, n = 6 987		
Q4 (>75)	1.00	1.00
Q3 (65-75)	1.30 (0.95-1.76)	1.27 (0.93-1.72)
Q2 (57-64)	1.25 (0.92-1.70)	1.17 (0.85-1.59)
Q1 (≤56)[b]	1.52 (1.13-2.05)	1.31 (0.96-1.78)
Dyslipidemia (<39 or previous diagnosis)	2.58 (1.52-4.36)	1.84 (1.02-3.30)
**Non-HDL-C** (mg/dL)<br>HDP, n = 406<br>Normotensive, n = 7 017		
Q1 (≤97)	1.00	1.00
Q2 (98-115)	0.98 (0.71-1.35)	0.93 (0.67-1.28)
Q3 (116-137)	1.32 (0.98-1.78)	1.25 (0.93-1.69)
Q4 (>137)[b]	1.41 (1.05-1.90)	1.27 (0.94-1.71)
Dyslipidemia (elevated[c] or previous diagnosis)	2.85 (1.88-4.33)	2.17 (1.38-3.42)
**Fasting glucose** (mg/dL)<br>HDP, n = 1 597<br>Normotensive, n = 28 327		
Q1 (≤74)	1.00	1.00
Q2 (75-80)	1.15 (0.99-1.34)	1.12 (0.96-1.31)
Q3 (81-85)	1.35 (1.16-1.57)	1.26 (1.09-1.47)
Q4 (86-99)[b]	1.29 (1.11-1.50)	1.13 (0.97-1.31)
Prediabetic ADA (100-109)	1.16 (0.75-1.79)	1.01 (0.65-1.56)
Prediabetic WHO (110-125)	1.42 (0.77-2.65)	1.19 (0.64-2.22)
Diabetic (≥126 or previous diagnosis)	3.18 (2.51-4.04)	1.89 (1.46-2.45)
**TyG index**<br>HDP, n = 1 5951 572<br>Normotensive, n = 27 933		
Q1 (≤6.06)	1.00	1.00
Q2 (6.07-6.37)	1.11 (0.95-1.29)	1.05 (0.90-1.23)
Q3 (6.38-6.71)	1.28 (1.10-1.48)	1.16 (1.00-1.35)
Q4 (>6.71)	1.47 (1.27-1.70)	1.20 (1.03-1.39)

ApoA1, apolipoprotein A1; ApoB, apolipoprotein B; CI, confidence interval; CRP, C-reactive protein; HDL_C, high-density lipoprotein cholesterol; HDP, hypertensive disorders of pregnancy; LDL-C, low-density lipoprotein cholesterol; OR, odds ratio; Q, quartile; TC, total cholesterol; TyG, triglyceride-glucose.

[a] Adjusted for continuous body mass index in early pregnancy, maternal age at delivery, calendar year of pregnancy, and maternal chronic hypertension. In analyses of inflammatory and lipid markers, the model additionally included adjustment for maternal diabetes mellitus. In analyses of inflammatory and glucose markers, the model additionally included adjustment for maternal dyslipidemia.

[b] The quartiles were mutually exclusive with respect to the categories based on clinical cut-off values.

[c] Age-specific clinical cutoffs were used for fasting triglycerides (>248 mg/dL [1-17 years] and >230 mg/dL [≥18 years]), TC (>228 mg/dL [1-14 years], >232 mg/dL [15-17 years], >236 mg/dL [18-30 years], and >267 mg/dL [31-50 years]), LDL-C (>155 mg/dL [1-17 years], >166 mg/dL [18-30 years], >182 mg/dL [31-50 years]), and non-HDL-C (>201 mg/dL [<1 year], >166 mg/dL [1-9 years], >155 mg/dL [10-17 years], >182 mg/dL [18-29 years], >197 mg/dL [30-49 years], >240 mg/dL [>50 years]).

© 2026 Qvick A et al. *JAMA Network Open.*

eTable 10. Associations between pregestational cardiometabolic biomarkers and risk of HDP after stratification by time between biomarker sampling and index pregnancy

	**Adjusted[a] OR (95 % CI)** Time tertile 1[b]	**Adjusted[a] OR (95 % CI)** Time tertile 2[b]	**Adjusted[a] OR (95 % CI)** Time tertile 3[b]
**Exposures**			
**CRP** (mg/dL)			
Q1 (≤0.20)	1.00	1.00	1.00
Q2 (0.21-0.40)	1.37 (0.97-1.92)	0.94 (0.68-1.30)	0.99 (0.76-1.30)
Q3 (0.41-0.60)	1.13 (0.77-1.67)	0.89 (0.62-1.27)	1.05 (0.79-1.40)
Q4 (>0.60)	0.93 (0.64-1.37)	1.08 (0.77-1.50)	0.88 (0.64-1-20)
**Leukocyte count** (/µL)			
Q1 (≤5300)	1.00	1.00	1.00
Q2 (5301-6300)	1.27 (0.88-1.84)	0.86 (0.59-1.25)	0.94 (0.68-1.30)
Q3 (6301-7500)	1.32 (0.92-1.91)	1.14 (0.80-1.62)	0.97 (0.70-1.34)
Q4 (>7500)	0.85 (0.57-1.28)	1.01 (0.70-1.44)	1.07 (0.79-1.44)
**Haptoglobin** (mg/dL)			
Q1 (≤800)	1.00	1.00	1.00
Q2 (801-909)	1.18 (0.82-1.70)	0.76 (0.54-1.06)	0.93 (0.69-1.24)
Q3 (910-1100)	1.65 (1.23-2.23)	1.03 (0.79-1.33)	1.20 (0.95-1.52)
Q4 (>1100)	1.26 (0.89-1.79)	1.28 (0.97-1.68)	1.12 (0.86-1.45)
**ApoA1** (mg/dL)			
Q4 (>160)	1.00	1.00	1.00
Q3 (146-160)	1.10 (0.64-1.89)	1.17 (0.66-2.06)	1.93 (1.13-3.30)
Q2 (133-145)	0.89 (0.51-1.58)	0.95 (0.53-1.72)	1.71 (1.00-2.93)
Q1 (≤132)	0.71 (0.39-1.30)	1.42 (0.82-2.46)	1.22 (0.70-2.12)
**ApoB** (mg/dL)			
Q1 (≤77)	1.00	1.00	1.00
Q2 (78-91)	0.92 (0.46-1.83)	1.05 (0.53-2.08)	1.46 (0.84-2.52)
Q3 (92-106)	1.15 (0.58-2.25)	1.58 (0.84-2.99)	1.61 (0.93-2.80)
Q4 (>106)	1.34 (0.70-2.56)	2.68 (1.46-4.91)	1.96 (1.15-3.34)
**ApoB/ApoA1 ratio**			
Q1 (≤0.51)	1.00	1.00	1.00
Q2 (0.52-0.62)	1.67 (0.80-3.49)	1.05 (0.52-2.10)	1.47 (0.80-2.69)
Q3 (0.63-0.75)	1.61 (0.76-3.40)	1.44 (0.75-2.77)	1.17 (0.63-2.16)
Q4 (>0.75)	1.38 (0.64-2.94)	2.00 (1.06-3.76)	1.59 (0.89-2.87)
**Fasting triglycerides** (mgl/dL)			
Q1 (≤53)	1.00	1.00	1.00
Q2 (54-71)	0.97 (0.73-1.28)	0.98 (0.76-1.26)	1.16 (0.92-1.47)
Q3 (72-97)	1.12 (0.85-1.48)	1.04 (0.81-1.34)	1.22 (0.97-1.54)
Q4 (>97)[c]	1.23 (0.93-1.64)	1.02 (0.80-1.32)	1.32 (1.05-1.65)
Dyslipidemia (elevated[d] or previous diagnosis)	2.56 (1.70-3.85)	2.11 (1.36-3.29)	1.73 (1.11-2.70)
**TC** (mg/dL)			
Q1 (≤164)	1.00	1.00	1.00
Q2 (165-179)	1.13 (0.82-1.56)	1.00 (0.76-1.32)	1.05 (0.83-1.32)
Q3 (180-201)	1.20 (0.90-1.62)	1.11 (0.86-1.44)	1.07 (0.86-1.34)
Q4 (>201)[c]	1.34 (1.00-1.79)	1.22 (0.95-1.57)	1.20 (0.96-1.50)
Dyslipidemia (elevated[d] or previous diagnosis)	2.01 (1.38-2.91)	1.66 (1.18-2.33)	1.52 (1.10-2.09)
**LDL-C** (mg/dL)			
Q1 (≤83)	1.00	1.00	1.00
Q2 (84-100)	1.66 (0.92-3.02)	1.11 (0.64-1.92)	1.06 (0.66-1.70)
Q3 (101-122)	0.84 (0.43-1.65)	1.17 (0.68-2.02)	1.20 (0.75-1.92)
Q4 (>122)[c]	1.32 (0.73-2.41)	1.57 (0.94-2.63)	1.37 (0.87-2.17)
Dyslipidemia (elevated[d] or previous diagnosis)	1.97 (0.80-4.89)	1.01 (0.30-3.47)	2.17 (0.89-5.31)
**HDL-C** (mg/dL)			
Q4 (>75)	1.00	1.00	1.00
Q3 (65-75)	1.23 (0.71-2.14)	1.07 (0.62-1.84)	1.52 (0.91-2.55)
Q2 (57-64)	0.94 (0.53-1.69)	1.17 (0.68-2.02)	1.36 (0.81-2.28)
Q1 (≤56)[c]	1.11 (0.62-1.96)	1.60 (0.95-2.69)	1.33 (0.80-2.23)
Dyslipidemia (<39 or previous diagnosis)	1.93 (0.80-4.64)	1.32 (0.38-4.58)	2.29 (0.83-6.30)

© 2026 Qvick A et al. *JAMA Network Open.*

eTable 10. Associations between pregestational cardiometabolic biomarkers and risk of HDP after stratification by time between biomarker sampling and index pregnancy

**Non-HDL-C** (mg/dL)			
Q1 (≤97)	1.00	1.00	1.00
Q2 (98-115)	1.21 (0.66-2.19)	0.71 (0.38-1.33)	0.94 (0.57-1.54)
Q3 (116-137)	0.97 (0.52-1.81)	1.40 (0.83-2.36)	1.33 (0.84-2.11)
Q4 (>137)[c]	1.11 (0.61-2.00)	1.63 (0.98-2.71)	1.10 (0.68-1.79)
Dyslipidemia (elevated[d] or previous diagnosis)	2.41 (1.12-5.21)	1.24 (0.45-3.41)	2.75 (1.37-5.53)
**Fasting glucose** (mg/dL)			
Q1 (≤74)	1.00	1.00	1.00
Q2 (75-80)	1.08 (0.80-1.47)	1.29 (0.98-1.70)	1.03 (0.81-1.31)
Q3 (81-85)	1.20 (0.89-1.62)	1.55 (1.19-2.03)	1.15 (0.91-1.46)
Q4 (86-99)[c]	1.27 (0.94-1.71)	1.15 (0.87-1.53)	1.11 (0.88-1.41)
Prediabetic ADA (100-109)	1.08 (0.46-2.54)	0.82 (0.32-2.05)	1.17 (0.63-2.16)
Prediabetic WHO (110-125)	-	1.75 (0.53-5.78)	1.40 (0.65-2.97)
Diabetic (≥126 or previous diagnosis)	1.88 (1.20-2.97)	2.37 (1.46-3.84)	1.91 (1.26-2.88)
**TyG** index			
Q1 (≤6.06)	1.00	1.00	1.00
Q2 (6.07-6.37)	0.96 (0.72-1.28)	1.07 (0.82-1.38)	1.13 (0.88-1.45)
Q3 (6.38-6.71)	1.10 (0.82-1.47)	1.05 (0.81-1.37)	1.32 (1.04-1.68)
Q4 (>6.71)	1.27 (0.96-1.69)	1.12 (0.86-1.46)	1.29 (1.02-1.64)

ApoA1, apolipoprotein A1; ApoB, apolipoprotein B; CI, confidence interval; CRP, C-reactive protein; HDL-C, high-density lipoprotein cholesterol; HDP, hypertensive disorders of pregnancy; LDL-C, low-density lipoprotein cholesterol; OR, odds ratio; TC, total cholesterol TyG, triglyceride-glucose.

[a] Adjusted for categorical body mass index in early pregnancy, maternal age at delivery, pregnancy calendar year, and maternal chronic hypertension. In analyses of inflammatory and lipid markers, the model additionally included adjustment for maternal diabetes mellitus. In analyses of inflammatory and glucose markers, the model additionally included adjustment for maternal dyslipidemia.

[b] Tertile limits in years for each biomarker. **CRP:** T1 <0.27, T2 = 0.27-0.69, T3 ≥0.70). **Leukocyte count:** T1 <3.3, T2 = 3.3-8.4, T3 ≥8.5. **Haptoglobin:** T1 <2.5, T2 = 2.5-6.7, T3 ≥6.8. **ApoA-1:** T1 <2.7, T2 = 2.7-6.6, T3 ≥6.7. **ApoB:** T1 <2.8, T2 = 2.8-6.8, T3 ≥6.9. **ApoB/apoA-1 ratio:** T1 <2.7, T2 = 2.7-6.7, T3 ≥6.8. **Fasting triglycerides:** T1 <2.4, T2 = 2.4-6.5, T3 ≥6.6. **TC:** T1 <2.4, T2 = 2.4-6.5, T3 ≥6.6. **LDL:** T1 <2.7, T2 = 2.7-6.5, T3 ≥6.6. **HDL:** T1 <2.7, T2 = 2.7-6.4, T3 ≥6.5. **Non-HDL:** T1 <2.7, T2 = 2.7-6.4, T3 ≥6.5. **Fasting glucose:** T1 <2.4, T2 = 2.4-6.5, T3 ≥6.6. **TyG:** T1 <2.4, T2 = 2.4-6.4, T3 ≥6.5.

[c] The quartiles were mutually exclusive with respect to the categories based on clinical cut-off values.

[d] Age-specific clinical cutoffs were used for fasting triglycerides (>248 mg/dL [1-17 years] and >230 mg/dL [≥18 years]), TC (>228 mg/dL [1-14 years], >232 mg/dL [15-17 years], >236 mg/dL [18-30 years], and >267 mg/dL [31-50 years]), LDL-C (>155 mg/dL [1-17 years], >166 mg/dL [18-30 years], >182 mg/dL [31-50 years]), and non-HDL-C (>201 mg/dL [<1 year], >166 mg/dL [1-9 years], >155 mg/dL [10-17 years], >182 mg/dL [18-29 years], >197 mg/dL [30-49 years], >240 mg/dL [>50 years]).

© 2026 Qvick A et al. *JAMA Network Open.*

eTable 11. Associations between pregestational cardiometabolic biomarkers and risk of HDP when restricting to occupational healthcare referrals

	Crude OR (95 % CI)	Adjusted[a] OR (95 % CI)
**Exposures**		
**CRP** (mg/dL)<br>HDP, n = 683<br>Normotensive, n = 10 962		
Q1 (≤0.20)	1.00	1.00
Q2 (0.21-0.40)	1.01 (0.82-1.25)	0.94 (0.75-1.17)
Q3 (0.41-0.60)	1.08 (0.85-1.36)	0.96 (0.75-1.22)
Q4 (>0.60)	1.06 (0.83-1.37)	0.95 (0.73-1.22)
**Leukocyte count** (/µL)<br>HDP, n = 758<br>Normotensive, n = 13 085		
Q1 (≤5300)	1.00	1.00
Q2 (5301-6300)	1.03 (0.83-1.28)	1.01 (0.82-1.25)
Q3 (6301-7500)	1.25 (1.02-1.54)	1.15 (0.94-1.42)
Q4 (>7500)	1.11 (0.90-1.37)	0.98 (0.79-1.21)
**Haptoglobin** (mg/dL)<br>HDP, n = 815<br>Normotensive, n = 13 637		
Q1 (≤800)	1.00	1.00
Q2 (801-909)	0.85 (0.67-1.08)	0.85 (0.67-1.08)
Q3 (910-1100)	1.41 (1.18-1.69)	1.30 (1.09-1.57)
Q4 (>1100)	1.47 (1.21-1.78)	1.19 (0.98-1.45)
**ApoA1** (mg/dL)<br>HDP, n = 193<br>Normotensive, n = 3 291		
Q4 (>160)	1.00	1.00
Q3 (146-160)	1.77 (1.15-2.73)	1.25 (0.80–1.95)
Q2 (133-145)	1.51 (0.96-2.37)	1.38 (0.88-2.17)
Q1 (≤132)	1.54 (1.00-2.37)	1.70 (1.10-2.64)
**ApoB** (mg/dL)<br>HDP, n = 193<br>Normotensive, n = 3 173		
Q1 (≤77)	1.00	1.00
Q2 (78-91)	1.28 (0.81-2.04)	1.30 (0.81-2.08)
Q3 (92-106)	1.48 (0.93–2.34)	1.57 (0.98-2.51)
Q4 (>106)	1.86 (1.21-2.86)	1.82 (1.16-2.84)
**ApoB/ApoA1 ratio**<br>HDP, n = 179<br>Normotensive, n = 2 967		
Q1 (≤0.51)	1.00	1.00
Q2 (0.52-0.62)	1.22 (0.76-1.96)	1.27 (0.79-2.06)
Q3 (0.63-0.75)	1.19 (0.74-1.92)	1.12 (0.69-1.81)
Q4 (>0.75)	1.69 (1.09-2.63)	1.48 (0.94-2.33)
**Fasting triglycerides** (mg/dL)<br>HDP, n = 1 075<br>Normotensive, n = 18 582		
Q1 (≤53)	1.00	1.00
Q2 (54-71)	1.07 (0.89-1.29)	1.03 (0.85-1.25)
Q3 (72-97)	1.30 (1.08-1.55)	1.20 (1.00-1.44)
Q4 (>97)[b]	1.44 (1.21-1.72)	1.24 (1.05-1.48)
Dyslipidemia (elevated[c] or previous diagnosis)	3.31 (2.54-4.32)	2.20 (1.64-2.95)
**TC** (mg/dL)<br>HDP, n = 1 079<br>Normotensive, n = 18 630		
Q1 (≤164)	1.00	1.00
Q2 (165-179)	1.14 (0.94-1.37)	1.13 (0.94-1.37)
Q3 (180-201)	1.10 (0.92-1.32)	1.08 (0.89-1.29)
Q4 (>201)[b]	1.33 (1.12-1.58)	1.28 (1.07-1.53)
Dyslipidemia (elevatedc or previous diagnosis)	2.08 (1.65-2.61)	1.69 (1.33-2.15)
**LDL-C** (mg/dL)<br>HDP, n = 211<br>Normotensive, n = 3 506		

© 2026 Qvick A et al. *JAMA Network Open.*

eTable 11. Associations between pregestational cardiometabolic biomarkers and risk of HDP when restricting to occupational healthcare referrals

Q1 (≤83)	1.00	1.00
Q2 (84-100)	1.54 (1.01-2.33)	1.49 (0.98-2.27)
Q3 (101-122)	1.03 (0.65-1.61)	1.03 (0.65-1.62)
Q4 (>122)[b]	1.47 (0.97-2.21)	1.37 (0.90-2.07)
Dyslipidemia (elevated[c] or previous diagnosis)	2.60 (1.40-4.85)	1.89 (0.94-3.80)
**HDL-C** (mg/dL)<br>HDP, n = 207<br>Normotensive, n = 3 481		
Q4 (>75)	1.00	1.00
Q3 (65-75)	1.53 (0.96-2.44)	1.46 (0.92-2.34)
Q2 (57-64)	1.65 (1.05-2.61)	1.47 (0.93-2.33)
Q1 (≤56)[b]	1.96 (1.27-3.03)	1.58 (1.00-2.58)
Dyslipidemia (<39 or previous diagnosis)	3.25 (1.65-6.41)	2.25 (1.07-4.75)
**Non-HDL-C** (mg/dL)<br>HDP, n = 211<br>Normotensive, n = 3 508		
Q1 (≤97)	1.00	1.00
Q2 (98-115)	0.98 (0.62-1.56)	0.94 (0.59-1.50)
Q3 (116-137)	1.38 (0.90-2.10)	1.29 (0.84-1.98)
Q4 (>137)[b]	1.32 (0.86-2.01)	1.17 (0.77-1.80)
Dyslipidemia (elevated[c] or previous diagnosis)	2.97 (1.75-5.03)	2.28 (1.28-4.04)
**Fasting glucose** (mg/dL)<br>HDP, n = 1 023<br>Normotensive, n = 18 074		
Q1 (≤74)	1.00	1.00
Q2 (75-80)	1.13 (0.93-1.38)	1.08 (0.89-1.32)
Q3 (81-85)	1.36 (1.13-1.64)	1.27 (1.05-1.53)
Q4 (86-99)[b]	1.27 (1.05-1.54)	1.12 (0.92-1.35)
Prediabetic ADA (100-109)	1.44 (0.89-2.34)	1.27 (0.78-2.06)
Prediabetic WHO (110-125)	1.60 (0.80-3.19)	1.30 (0.65-2.62)
Diabetic (≥126 or previous diagnosis)	3.04 (2.27-4.05)	1.86 (1.36-2.54)
**TyG index**<br>HDP, n = 1 001<br>Normotensive, n = 17 701		
Q1 (≤6.06)	1.00	1.00
Q2 (6.07-6.37)	1.09 (0.89-1.32)	1.03 (0.85-1.26)
Q3 (6.38-6.71)	1.36 (1.13-1.64)	1.23 (1.02-1.49)
Q4 (>6.71)	1.50 (1.25-1.80)	1.23 (1.02-1.48)

ApoA1, apolipoprotein A1; ApoB, apolipoprotein B; CI, confidence interval; CRP, C-reactive protein; HDL-C, high-density lipoprotein cholesterol; HDP, hypertensive disorders of pregnancy; LDL-C, low-density lipoprotein cholesterol; OR, odds ratio; Q, quartile; TC, total cholesterol; TyG, triglyceride-glucose.

Individuals referred to blood sampling by primary health care were excluded in these analyses. These constituted 23-34% of the participants included in analyses of all biomarkers, except for leukocyte count where the proportion was 61%.

[a] Adjusted for categorical body mass index in early pregnancy, maternal age at delivery, pregnancy calendar year, and maternal chronic hypertension. In analyses of inflammatory and lipid markers, the model additionally included adjustment for maternal diabetes mellitus. In analyses of inflammatory and glucose markers, the model additionally included adjustment for maternal dyslipidemia.

[b] The quartiles were mutually exclusive with respect to the categories based on clinical cut-off values.

[c] Age-specific clinical cutoffs were used for fasting triglycerides (>248 mg/dL [1-17 years] and >230 mg/dL [≥18 years]), TC (>228 mg/dL [1-14 years], >232 mg/dL [15-17 years], >236 mg/dL [18-30 years], and >267 mg/dL [31-50 years]), LDL-C (>155 mg/dL [1-17 years], >166 mg/dL [18-30 years], >182 mg/dL [31-50 years]), and non-HDL-C (>201 mg/dL [<1 year], >166 mg/dL [1-9 years], >155 mg/dL [10-17 years], >182 mg/dL [18-29 years], >197 mg/dL [30-49 years], >240 mg/dL [>50 years]).

© 2026 Qvick A et al. *JAMA Network Open*.

eTable 12. Associations between pregestational cardiometabolic biomarkers and risk of HDP when restricting to individuals with singleton pregnancies

	Crude OR (95 % CI)	Adjusted[a] OR (95 % CI)
**Exposures**		
**CRP** (mg/dL)<br>HDP, n = 1 049<br>Normotensive, n = 17 623		
Q1 (≤0.20)	1.00	1.00
Q2 (0.21-0.40)	1.15 (0.98-1.36)	1.05 (0.88-1.24)
Q3 (0.41-0.60)	1.13 (0.94-1.36)	0.99 (0.82-1.20)
Q4 (>0.60)	1.09 (0.90-1.33)	0.98 (0.80-1.19)
**Leukocyte count** (/µL)<br>HDP, n = 797<br>Normotensive, n = 14 053		
Q1 (≤5300)	1.00	1.00
Q2 (5301-6300)	1.06 (0.86-1.31)	1.04 (0.85-1.29)
Q3 (6301-7500)	1.27 (1.04-1.55)	1.17 (0.95-1.43)
Q4 (>7500)	1.16 (0.95-1.42)	1.02 (0.83-1.26)
**Haptoglobin** (mg/dL)<br>HDP, n = 1 132<br>Normotensive, n = 20 738		
Q1 (≤800)	1.00	1.00
Q2 (810-909)	0.91 (0.75-1.10)	0.89 (0.73-1.08)
Q3 (910-1100)	1.34 (1.16-1.56)	1.25 (1.07-1.45)
Q4 (>1100)	1.44 (1.22-1.70)	1.17 (0.99-1.39)
**ApoA1** (mg/dL)<br>HDP, n = 337<br>Normotensive, n = 6 142		
Q4 (>160)	1.00	1.00
Q3 (146-160)	1.47 (1.07-2.01)	1.45 (1.06-2.00)
Q2 (133-145)	1.26 (0.91-1.75)	1.21 (0.87-1.68)
Q1 (≤132)	1.26 (0.91-1.75)	1.09 (0.78-1.52)
**ApoB** (mg/dL)<br>HDP, n = 302<br>Normotensive, n = 5 606		
Q1 (≤77)	1.00	1.00
Q2 (78-91)	1.17 (0.81-1.68)	1.22 (0.84-1.77)
Q3 (92-106)	1.36 (0.95-1.94)	1.47 (1.02-2.12)
Q4 (>106)	1.91 (1.36-2.66)	1.95 (1.38-2.75)
**ApoB/ApoA1 ratio**<br>HDP, n = 257<br>Normotensive, n = 4 633		
Q1 (≤0.51)	1.00	1.00
Q2 (0.52-0.62)	1.43 (0.96-2.12)	1.52 (1.02-2.26)
Q3 (0.63-0.75)	1.49 (1.01-2.20)	1.43 (0.97-2.13)
Q4 (>0.75)	1.88 (1.30-2.73)	1.72 (1.17-2.53)
**Fasting triglycerides** (mg/dL)<br>HDP, n = 1 569<br>Normotensive, n = 28 459		
Q1 (≤53)	1.00	1.00
Q2 (54-71)	1.06 (0.91-1.23)	1.02 (0.88-1.19)
Q3 (72-97)	1.22 (1.05-1.41)	1.13 (0.97-1.31)
Q4 (>97)[b]	1.38 (1.20-1.59)	1.18 (1.02-1.37)
Dyslipidemia (elevated[c] or previous diagnosis)	3.15 (2.50-3.96)	2.12 (1.65-2.73)
**TC** (mg/dL)<br>HDP, n = 1 576<br>Normotensive, n = 28 521		
Q1 (≤164)	1.00	1.00
Q2 (165-179)	1.01 (0.86-1.18)	1.01 (0.86-1.19)
Q3 (180-201)	1.11 (0.96-1.29)	1.10 (0.95-1.28)
Q4 (>201)[b]	1.27 (1.10-1.46)	1.24 (1.07-1.43)
Dyslipidemia (elevated[c] or previous diagnosis)	2.07 (1.71-2.50)	1.70 (1.40-2.08)
**LDL-C** (mg/dL)<br>HDP, n = 392<br>Normotensive, n = 6 874		

© 2026 Qvick A et al. *JAMA Network Open.*

eTable 12. Associations between pregestational cardiometabolic biomarkers and risk of HDP when restricting to individuals with singleton pregnancies

Q1 (≤83)	1.00	1.00
Q2 (84-100)	1.22 (0.90-1.66)	1.19 (0.87-1.63)
Q3 (101-122)	1.06 (0.78-1.46)	1.05 (0.76-1.45)
Q4 (>122)[b]	1.54 (1.15-2.07)	1.41 (1.04-1.90)
Dyslipidemia (elevated[c] or previous diagnosis)	2.43 (1.47-4.02)	1.80 (1.03-3.15)
**HDL-C** (mgl/dL)<br>HDP, n = 387<br>Normotensive, n = 6 860		
Q4 (>75)	1.00	1.00
Q3 (65-75)	1.40 (1.01-1.90)	1.36 (0.99-1.87)
Q2 (57-64)	1.31 (0.95-1.80)	1.23 (0.89-1.70)
Q1 (≤56)[b]	1.60 (1.17-2.17)	1.39 (1.01-1.90)
Dyslipidemia (<39 or previous diagnosis)	2.86 (1.68-4.87)	2.10 (1.17-3.76)
**Non-HDL-C** (mg/dL)<br>HDP, n = 391<br>Normotensive, n = 6 890		
Q1 (≤97)	1.00	1.00
Q2 (98-115)	0.91 (0.66-1.27)	0.88 (0.63-1.23)
Q3 (116-137)	1.31 (0.97-1.78)	1.24 (0.91-1.68)
Q4 (>137)[b]	1.42 (1.05-1.92)	1.27 (0.94-1.72)
Dyslipidemia (elevated[c] or previous diagnosis)	2.90 (1.90-4.44)	2.20 (1.39-3.48)
**Fasting glucose** (mg/dL)<br>HDP, n = 1 518<br>Normotensive, n = 27 873		
Q1 (≤74)	1.00	1.00
Q2 (75-80)	1.14 (0.98-1.34)	1.11 (0.95-1.31)
Q3 (81-85)	1.40 (1.19-1.62)	1.30 (1.12-1.52)
Q4 (86-99)[b]	1.28 (1.10-1.50)	1.13 (0.96-1.32)
Prediabetic ADA (100-109)	1.06 (0.67-1.69)	0.93 (0.59-1.48)
Prediabetic WHO (110-125)	1.38 (0.72-2.63)	1.15 (0.60-2.21)
Diabetic (≥126 or previous diagnosis)	3.21 (2.52-4.10)	1.95 (1.50-2.54)
**TyG index**<br>HDP, n = 1 494<br>Normotensive, n = 27 484		
Q1 (≤6.06)	1.00	1.00
Q2 (6.07-6.37)	1.10 (0.94-1.29)	1.05 (0.90-1.23)
Q3 (6.38-6.71)	1.25 (1.08-1.46)	1.14 (0.97-1.33)
Q4 (>6.71)	1.49 (1.29-1.73)	1.22 (1.04-1.42)

ApoA1, apolipoprotein A1; ApoB, apolipoprotein B; CI, confidence interval; CRP, C-reactive protein; HDL-C, high-density lipoprotein cholesterol; HDP, hypertensive disorders of pregnancy; LDL-C, low-density lipoprotein cholesterol; OR, odds ratio; Q, quartile; TC, total cholesterol; TyG, triglyceride-glucose.

[a] Adjusted for categorical body mass index in early pregnancy, maternal age at delivery, pregnancy calendar year, and maternal chronic hypertension. In analyses of inflammatory and lipid markers, the model additionally included adjustment for maternal diabetes mellitus. In analyses of inflammatory and glucose markers, the model additionally included adjustment for maternal dyslipidemia.

[b] The quartiles were mutually exclusive with respect to the categories based on clinical cut-off values.

[c] Age-specific clinical cutoffs were used for fasting triglycerides (>248 mg/dL [1-17 years] and >230 mg/dL [≥18 years]), TC (>228 mg/dL [1-14 years], >232 mg/dL [15-17 years], >236 mg/dL [18-30 years], and >267 mg/dL [31-50 years]), LDL-C (>155 mg/dL [1-17 years], >166 mg/dL [18-30 years], >182 mg/dL [31-50 years]), and non-HDL-C (>201 mg/dL [<1 year], >166 mg/dL [1-9 years], >155 mg/dL [10-17 years], >182 mg/dL [18-29 years], >197 mg/dL [30-49 years], >240 mg/dL [>50 years]).

© 2026 Qvick A et al. *JAMA Network Open.*

eTable 13. Associations between pregestational cardiometabolic biomarkers and risk of HDP when restricting to individuals with no prophylactic prenatal acetyl-salicylic acid use during index pregnancy

	Crude OR (95 % CI)	Adjusted[a] OR (95 % CI)
**Exposures**		
**CRP** (mg/dL)<br>HDP, n = 1 091<br>Normotensive, n = 17 871		
Q1 (≤0.20)	1.00	1.00
Q2 (0.21-0.40)	1.14 (0.97-1.34)	1.03 (0.87-1.22)
Q3 (0.41-0.60)	1.14 (0.95-1.36)	1.00 (0.83-1.20)
Q4 (>0.60)	1.08 (0.89-1.31)	0.97 (0.80-1.18)
**Leukocyte count** (/μL)<br>HDP, n = 824<br>Normotensive, n = 14 212		
Q1 (≤5300)	1.00	1.00
Q2 (5301-6300)	1.01 (0.82-1.24)	0.99 (0.81-1.21)
Q3 (6301-7500)	1.21 (0.99-1.47)	1.11 (0.91-1.36)
Q4 (>7500)	1.10 (0.90-1.34)	0.97 (0.79-1.19)
**Haptoglobin** (mg/dL)<br>HDP, n = 1 186<br>Normotensive, n = 21 022		
Q1 (≤800)	1.00	1.00
Q2 (801-909)	0.95 (0.79-1.14)	0.93 (0.77-1.12)
Q3 (910-1100)	1.34 (1.16-1.56)	1.25 (1.07-1.45)
Q4 (>1100)	1.48 (1.26-1.74)	1.21 (1.02-1.42)
**ApoA1** (mg/dL)<br>HDP, n = 348<br>Normotensive, n = 6 238		
Q4 (>160)	1.00	1.00
Q3 (146-160)	1.43 (1.05-1.95)	1.40 (1.03-1.92)
Q2 (133-145)	1.25 (0.91-1.72)	1.19 (0.86-1.64)
Q1 (≤132)	1.26 (0.92-1.73)	1.09 (0.78-1.51)
**ApoB** (mg/dL)<br>HDP, n = 316<br>Normotensive, n = 5 698		
Q1 (≤77)	1.00	1.00
Q2 (78-91)	1.10 (0.78-1.59)	1.16 (0.81-1.66)
Q3 (92-106)	1.33 (0.94-1.88)	1.43 (1.01-2.04)
Q4 (>106)	1.84 (1.33-2.55)	1.88 (1.34-2.63)
**ApoB/ApoA1 ratio**<br>HDP, n = 268<br>Normotensive, n = 4 714		
Q1 (≤0.51)	1.00	1.00
Q2 (0.52-0.62)	1.32 (0.90-1.94)	1.39 (0.95-2.05)
Q3 (0.63-0.75)	1.42 (0.97-2.06)	1.36 (0.93-1.99)
Q4 (>0.75)	1.75 (1.22-2.52)	1.61 (1.11-2.34)
**Fasting triglycerides** (mg/dL)<br>HDP, n = 1 641<br>Normotensive, n = 28 845		
Q1 (≤53)	1.00	1.00
Q2 (54-71)	1.08 (0.93-1.25)	1.04 (0.90-1.20)
Q3 (72-97)	1.21 (1.05-1.40)	1.13 (0.97-1.30)
Q4 (>97)[b]	1.37 (1.19-1.58)	1.18 (1.03-1.37)
Dyslipidemia (elevated[c] or previous diagnosis)	3.04 (2.43-3.82)	2.09 (1.63-2.68)
**TC** (mg/dL)<br>HDP, n = 1 650<br>Normotensive, n = 28 909		
Q1 (≤164)	1.00	1.00
Q2 (165-179)	1.03 (0.89-1.21)	1.04 (0.89-1.21)
Q3 (180-201)	1.11 (0.96-1.28)	1.10 (0.95-1.27)
Q4 (>201)[b]	1.25 (1.09-1.44)	1.21 (1.05-1.40)
Dyslipidemia (elevated[c] or previous diagnosis)	2.02 (1.68-2.44)	1.68 (1.38-2.05)
**LDL-C** (mg/dL)<br>HDP, n = 406<br>Normotensive, n = 6 982		

eTable 13. Associations between pregestational cardiometabolic biomarkers and risk of HDP when restricting to individuals with no prophylactic prenatal acetyl-salicylic acid use during index pregnancy

Q1 (≤83)	1.00	1.00
Q2 (84-100)	1.26 (0.93-1.71)	1.23 (0.91-1.66)
Q3 (101-122)	1.08 (0.79-1.47)	1.06 (0.78-1.45)
Q4 (>122)[b]	1.53 (1.15-2.05)	1.40 (1.05-1.89)
Dyslipidemia (elevated[c] or previous diagnosis)	2.21 (1.32-3.68)	1.79 (1.03-3.12)
**HDL-C** (mg/dL)<br>HDP, n = 401<br>Normotensive, n = 6 967		
Q4 (>75)	1.00	1.00
Q3 (65-75)	1.28 (0.94-1.74)	1.25 (0.92-1.70)
Q2 (57-64)	1.25 (0.92-1.70)	1.17 (0.86-1.60)
Q1 (≤56)[b]	1.52 (1.13-2.05)	1.32 (0.97-1.79)
Dyslipidemia (<39 or previous diagnosis)	2.43 (1.42-4.16)	1.97 (1.10-3.51)
**Non-HDL-C** (mg/dL)<br>HDP, n = 405<br>Normotensive, n = 6 997		
Q1 (≤97)	1.00	1.00
Q2 (98-115)	0.98 (0.71-1.35)	0.95 (0.69-1.31)
Q3 (116-137)	1.31 (0.97-1.77)	1.24 (0.91-1.67)
Q4 (>137)[b]	1.41 (1.05-1.90)	1.26 (0.93-1.70)
Dyslipidemia (elevated[c] or previous diagnosis)	2.77 (1.81-4.22)	2.27 (1.44-3.56)
**Fasting glucose** (mg/dL)<br>HDP, n = 1 589<br>Normotensive, n = 28 247		
Q1 (≤74)	1.00	1.00
Q2 (75-80)	1.15 (0.99-1.34)	1.12 (0.96-1.31)
Q3 (81-85)	1.35 (1.16-1.56)	1.26 (1.09-1.47)
Q4 (86-99)[b]	1.28 (1.10-1.49)	1.13 (0.97-1.32)
Prediabetic ADA (100-109)	1.11 (0.71-1.73)	0.98 (0.63-1.53)
Prediabetic WHO (110-125)	1.44 (0.78-2.68)	1.21 (0.65-2.27)
Diabetic (≥126 or previous diagnosis)	3.18 (2.51-4.04)	1.96 (1.52-2.54)
**TyG index**<br>HDP, n = 1 564<br>Normotensive, n = 27 855		
Q1 (≤6.06)	1.00	1.00
Q2 (6.07-6.37)	1.11 (0.95-1.29)	1.06 (0.91-1.23)
Q3 (6.38-6.71)	1.26 (1.09-1.47)	1.15 (0.99-1.33)
Q4 (>6.71)	1.46 (1.27-1.69)	1.20 (1.04-1.40)

ApoA1, apolipoprotein A1; ApoB, apolipoprotein B; CI, confidence interval; CRP, C-reactive protein; HDL-C, high-density lipoprotein cholesterol; HDP, hypertensive disorders of pregnancy; LDL-C, low-density lipoprotein cholesterol; OR, odds ratio; Q, quartile; TC, total cholesterol; TyG. triglyceride-glucose.

[a] Adjusted for categorical body mass index in early pregnancy, maternal age at delivery, pregnancy calendar year, and maternal chronic hypertension. In analyses of inflammatory and lipid markers, the model additionally included adjustment for maternal diabetes mellitus. In analyses of inflammatory and glucose markers, the model additionally included adjustment for maternal dyslipidemia.

[b] The quartiles were mutually exclusive with respect to the categories based on clinical cut-off values.

[c] Age-specific clinical cutoffs were used for fasting triglycerides (>248 mg/dL [1-17 years] and >230 mg/dL [≥18 years]), TC (>228 mg/dL [1-14 years], >232 mg/dL [15-17 years], >236 mg/dL [18-30 years], and >267 mg/dL [31-50 years]), LDL-C (>155 mg/dL [1-17 years], >166 mg/dL [18-30 years], >182 mg/dL [31-50 years]), and non-HDL-C (>201 mg/dL [<1 year], >166 mg/dL [1-9 years], >155 mg/dL [10-17 years], >182 mg/dL [18-29 years], >197 mg/dL [30-49 years], >240 mg/dL [>50 years]).

© 2026 Qvick A et al. *JAMA Network Open.*

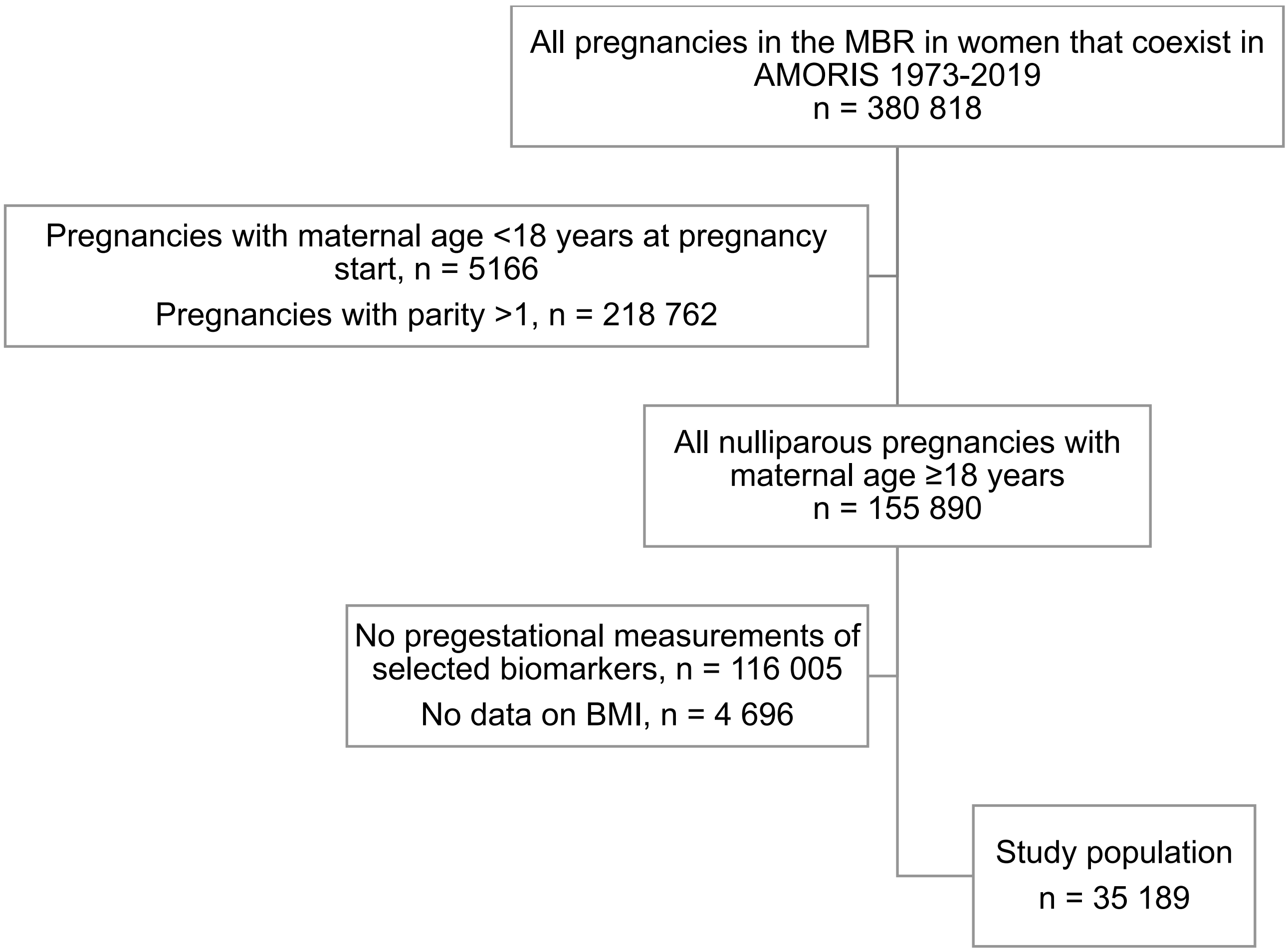

eFigure 1. Flowchart of participant inclusion in the study
AMORIS, Apolipoprotein-related MOrtality RISk cohort; BMI, body mass index; MBR, Swedish Medical Birth Register.

© 2026 Qvick A et al. *JAMA Network Open*.

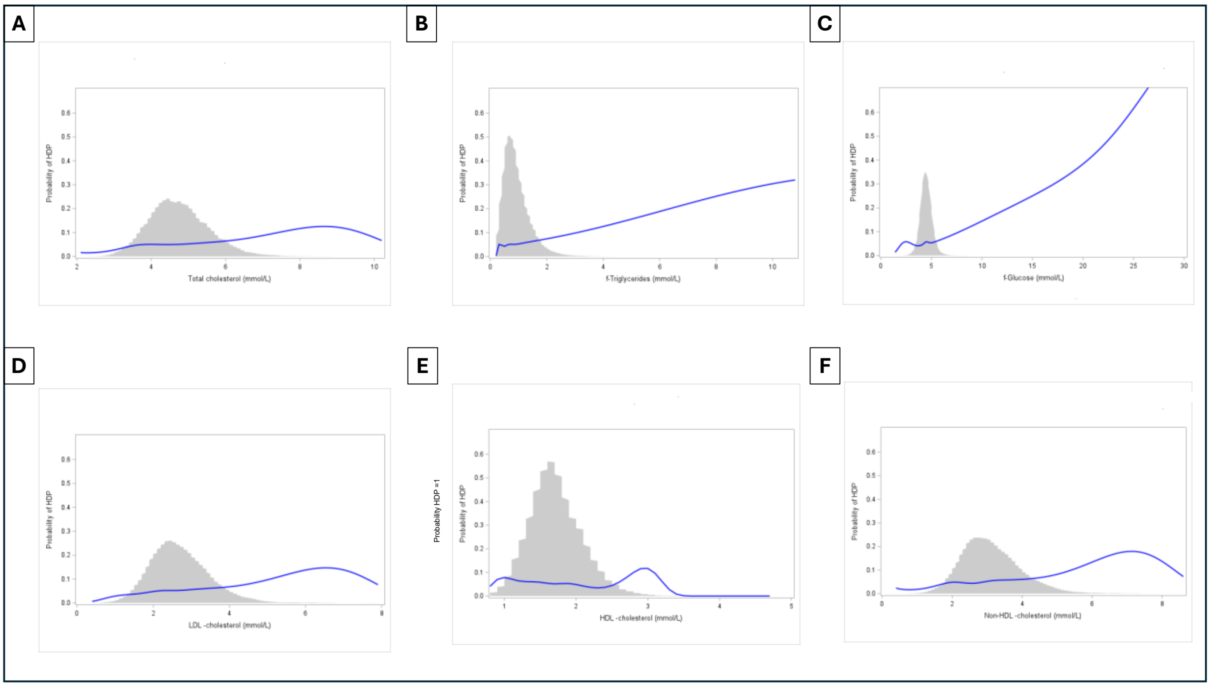

eFigure 2. Predicted probability of HDP across the range of six selected biomarkers (panel A-F), modelled using restricted cubic splines with knots at the 5th, 27.5th, 50th, 72.5th and 95th percentiles. The shaded area represents the distribution of values in the studied population. Models are adjusted for the same covariates as in the main analyses. For participants with diagnosed dyslipidemia or diabetes but missing biomarker values, the median value among those above the clinical cutoff was imputed. The proportion of imputed values was as follows: TC 6.0%, LDL 2.9%, HDL 2.4%, non-HDL 5.6%, Fasting glucose 4.7%, and Fasting triglycerides 2.7%.

<u>Cutoff values (Q1-Q4, clinical cutoff)</u>

TC (mg/dL):	≤164	165-179	180-201	>201	*
Fasting triglycerides (mg/dL):	≤53	54-71	72-97	>97	*
Fasting glucose (mg/dL):	≤74	75-80	81-85	>85	**
LDL-C (mg/dL):	≤83	84-100	101-122	>122	*
HDL-C (mg/dL):	≤56	57-64	65-75	>75	<39
Non-HDL-C (mg/dL):	≤97	98-115	116-137	>137	*

* Age-specific clinical cutoffs were used for fasting triglycerides (>248 mg/dL [1-17 years] and >230 mg/dL [≥18 years]), TC (>228 mg/dL [1-14 years], >232 mg/dL [15-17 years], >236 mg/dL [18-30 years], and >267 mg/dL [31-50 years]), LDL-C (>155 mg/dL [1-17 years], >166 mg/dL [18-30 years], >182 mg/dL [31-50 years]), and non-HDL-C (>201 mg/dL [<1 year], >166 mg/dL [1-9 years], >155 mg/dL [10-17 years], >182 mg/dL [18-29 years], >197 mg/dL [30-49 years], >240 mg/dL [>50 years]).

** 'Prediabetes ADA' (100-109 mg/dL), 'Prediabetes WHO' (110-125 mg/dL), and 'Diabetes' (≥126 mg/dL).

HDL-C, high-density lipoprotein cholesterol; HDP, hypertensive disorders of pregnancy; LDL-C, low-density lipoprotein cholesterol; OR, odds ratio; Q, quartile; TC, total cholesterol.

© 2026 Qvick A et al. *JAMA Network Open*.

eTable 14. Associations assessed through linear regression between pregestational cardiometabolic biomarkers and risk of HDP for biomarkers with a visual linearity in the restricted cubic splines models

	Crude OR (95 % CI)	Adjusted[a] OR (95 % CI)
**Exposures**		
**Fasting triglycerides**<br>HDP, n = 1 652<br>Normotensive, n = 28 932	1.35 (1.24-1.47)	1.18 (1.08-1.29)
**TC**<br>HDP, n = 1 661<br>Normotensive, n = 28 996	1.22 (1.15-1.29)	1.18 (1.12-1.26)
**LDL-C**<br>HDP, n = 408<br>Normotensive, n = 7 007	1.28 (1.14-1.44)	1.22 (1.08-1.37)
**Non-HDL-C**<br>HDP, n = 407<br>Normotensive, n = 7 022	1.31 (1.17-1.46)	1.22 (1.09-1.36)

CI, confidence interval; HDL-C, high-density lipoprotein cholesterol; HDP, hypertensive disorders of pregnancy; LDL-C, low-density lipoprotein cholesterol; OR, odds ratio; TC, total cholesterol.

[a] Adjusted for categorical body mass index in early pregnancy, maternal age at delivery, pregnancy calendar year, and maternal chronic hypertension. In analyses of inflammatory and lipid markers, the model additionally included adjustment for maternal diabetes mellitus. In analyses of inflammatory and glucose markers, the model additionally included adjustment for maternal dyslipidemia.

© 2026 Qvick A et al. *JAMA Network Open*.

eMethods.

Briefly, levels of f-triglycerides, f-glucose, and TC were measured with enzymatic techniques. Serum concentrations of non-sensitive CRP, haptoglobin, and ApoA1 and ApoB were analyzed by immunoturbidimetric assay. Leukocyte count was assessed by hematology analyzers. Blood LDL-C and HDL-C were calculated according to the previously validated Jungner formula[1 2]: LDL-C = 0.48 + 0.99 x TC – 0.23 x triglycerides – 1.58 x apoA-1; HDL-C = TC – 0.45 x triglycerides – LDL-C, or the Friedewald formula: LDL-C = (TC – HDL-C – triglycerides)/5. Non-HDL-C was obtained through subtracting HDL-C from TC (non-HDL-C = TC [mmol/L] – HDL-C [mmol/L]-). To calculate TyG index, the formula used was the natural logarithm (Ln) of the product of plasma glucose and triglycerides (Ln (triglycerides [mg/dL] x glucose [mg/dL])/2)).

eReferences.

1. Talmud PJ, Hawe E, Miller GJ, et al. Nonfasting apolipoprotein B and triglyceride levels as a useful predictor of coronary heart disease risk in middle-aged UK men. *Arterioscler Thromb Vasc Biol* 2002;22(11):1918-23. doi: 10.1161/01.atv.0000035521.22199.c7
2. Walldius G, Jungner I, Holme I, et al. High apolipoprotein B, low apolipoprotein A-I, and improvement in the prediction of fatal myocardial infarction (AMORIS study): a prospective study. *Lancet* 2001;358(9298):2026-33. doi: 10.1016/S0140-6736(01)07098-2

© 2026 Qvick A et al. *JAMA Network Open*.
