# Supplementary material for: Pregestational Cardiometabolic Biomarkers and Future Hypertensive Disorders of Pregnancy
Source: JAMA Netw Open. 2026 Apr 30;9(4):e2610037. doi: 10.1001/jamanetworkopen.2026.10037 (PMC13133693; doi:10.1001/jamanetworkopen.2026.10037)
Supplement: Supplement 2. — Data Sharing Statement [file jamanetwopen-e2610037-s002.pdf]

Data Sharing Statement

Qvick. Pregestational Cardiometabolic Biomarkers and Future Hypertensive Disorders of Pregnancy. *JAMA Netw Open*. Published April 30, 2026.
doi:10.1001/jamanetworkopen.2026.10037

Data
Data available: No
